# Integrated Single-cell Multiomic Analysis of HIV Latency Reversal Reveals Novel Regulators of Viral Reactivation

**DOI:** 10.1093/gpbjnl/qzae003

**Published:** 2024-06-20

**Authors:** Manickam Ashokkumar, Wenwen Mei, Jackson J Peterson, Yuriko Harigaya, David M Murdoch, David M Margolis, Caleb Kornfein, Alex Oesterling, Zhicheng Guo, Cynthia D Rudin, Yuchao Jiang, Edward P Browne

**Affiliations:** Department of Medicine, University of North Carolina at Chapel Hill, Chapel Hill, NC 27599, USA; HIV Cure Center, University of North Carolina at Chapel Hill, Chapel Hill, NC 27599, USA; Department of Biostatistics, University of North Carolina at Chapel Hill, Chapel Hill, NC 27599, USA; HIV Cure Center, University of North Carolina at Chapel Hill, Chapel Hill, NC 27599, USA; Department of Microbiology and Immunology, University of North Carolina at Chapel Hill, Chapel Hill, NC 27599, USA; Department of Genetics, University of North Carolina at Chapel Hill, Chapel Hill, NC 27599, USA; Department of Medicine, Duke University, Durham, NC 27708, USA; Department of Medicine, University of North Carolina at Chapel Hill, Chapel Hill, NC 27599, USA; HIV Cure Center, University of North Carolina at Chapel Hill, Chapel Hill, NC 27599, USA; Department of Microbiology and Immunology, University of North Carolina at Chapel Hill, Chapel Hill, NC 27599, USA; Department of Computer Science, Duke University, Durham, NC 27708, USA; Department of Computer Science, Duke University, Durham, NC 27708, USA; Department of Computer Science, Duke University, Durham, NC 27708, USA; Department of Computer Science, Duke University, Durham, NC 27708, USA; Department of Statistics, Texas A&M University, College Station, TX 77843, USA; Department of Biology, Texas A&M University, College Station, TX 77843, USA; Department of Biomedical Engineering, Texas A&M University, College Station, TX 77843, USA; Department of Medicine, University of North Carolina at Chapel Hill, Chapel Hill, NC 27599, USA; HIV Cure Center, University of North Carolina at Chapel Hill, Chapel Hill, NC 27599, USA; Department of Microbiology and Immunology, University of North Carolina at Chapel Hill, Chapel Hill, NC 27599, USA

**Keywords:** HIV latency reversal, Primary CD4^+^ T cell, Single-cell multiomics, HIV-regulating factor, Machine learning

## Abstract

Despite the success of antiretroviral therapy, human immunodeficiency virus (HIV) cannot be cured because of a reservoir of latently infected cells that evades therapy. To understand the mechanisms of HIV latency, we employed an integrated single-cell RNA sequencing (scRNA-seq) and single-cell assay for transposase-accessible chromatin with sequencing (scATAC-seq) approach to simultaneously profile the transcriptomic and epigenomic characteristics of ∼ 125,000 latently infected primary CD4^+^ T cells after reactivation using three different latency reversing agents. Differentially expressed genes and differentially accessible motifs were used to examine transcriptional pathways and transcription factor (TF) activities across the cell population. We identified cellular transcripts and TFs whose expression/activity was correlated with viral reactivation and demonstrated that a machine learning model trained on these data was 75%–79% accurate at predicting viral reactivation. Finally, we validated the role of two candidate HIV-regulating factors, FOXP1 and GATA3, in viral transcription. These data demonstrate the power of integrated multimodal single-cell analysis to uncover novel relationships between host cell factors and HIV latency.

## Introduction

The formation of a latently infected reservoir in CD4^+^ T cells is a key barrier to an human immunodeficiency virus (HIV) cure [1,2]. The reservoir is highly stable and long lived, and is not eliminated by current antiretroviral therapy [3]. Additionally, clonal expansion *in vivo* counteracts gradual erosion of infected cells [4–8]. A key mechanism in the formation of this reservoir is the phenomenon of viral latency, in which HIV transcription is reversibly silenced post integration, allowing it to evade the host immune defenses. Sporadic reactivation of these cells generates “blips” of viremia during therapy [9] and seeds rapid rebound upon interruption of therapy [10,11]. Current cure strategies for HIV involve transiently inducing viral gene expression in latent proviruses using latency reversing agents (LRAs), followed by immune clearance of the reactivated cells. However, this approach has thus far only achieved limited success [12–16]. Reactivation of latently infected cells with existing LRAs is inefficient, with typically ∼ 10% of replication-competent proviruses being reactivated in patient-derived cells, even with “potent” LRAs [17]. For the LRA/clearance approach to be successful, broad reactivation of the reservoir will be required. This inefficiency of latency reversal with LRAs is likely due to a combination of stochastic processes that regulate viral gene expression, the existence of multiple layers of repression to HIV gene expression, and the heterogeneity in both the cellular environment and proviral integration sites in infected cells. Fully defining the nature of these repressive mechanisms is urgently needed to allow the development of broader acting LRAs or approaches with a combination of different LRAs. Prior work indicates that silencing of HIV in CD4^+^ T cells involves the combined effects of low levels of positive transcription factors (TFs), such as nuclear factor kappa B (NF-κB) and activating protein-1 (AP-1) [18–20], active repression by cellular factors (*e.g.*, NELF and DSIF) [21,22], sequestration of the elongation complex P-TEFb (cyclinT1/CDK9) [23], and histone modifications that create a repressive heterochromatin environment around the virus promoter [23–27]. Nevertheless, the regulation of HIV gene expression remains incompletely understood, and additional mechanisms likely exist that will need to be fully characterized.

Gene expression is regulated by TFs that bind to promoter or enhancer regions near the gene, and mediate chromatin remodeling of these regions [28], during which nucleosomes are removed, repositioned, or modified by covalent molecules such as acetylation or methylation [29]. This remodeling then allows recruitment of additional TFs, as well as RNA polymerase II (RNA Pol II) and transcriptional elongation factors to promoters and enhancers. Thus, TF binding, chromatin remodeling, and RNA transcription are key regulatory steps in cellular gene expression that act in a coordinated fashion to determine the expression level for each gene. However, fully understanding how these processes interact to regulate gene activities in individual cells has been difficult due to the technical limitations of profiling individual cells. Recent advances in multimodal single-cell analyses now allow concurrent observation of both the RNA levels for a given gene and the accessibility of the chromatin associated with this gene in single cells [30]. These approaches have led to fundamental new insights into the regulation of gene expression. Thus, in this study, we used an integrated single-cell RNA sequencing (scRNA-seq) and single-cell assay for transposase-accessible chromatin with sequencing (scATAC-seq) approach to characterize transcriptomic and epigenomic profiles from same cells in both a cell line model of HIV latency and a primary CD4^+^ T cell latency model to uncover the networks of transcriptional regulators that control HIV gene expression in response to hallmark LRAs. By correlating HIV viral RNA (vRNA) levels with individual cellular transcripts from scRNA-seq, as well as with chromatin peak dynamics and TF deviation scores inferred from scATAC-seq peak accessibility, we revealed several novel candidate regulators of HIV gene expression. Finally, by using short hairpin RNA (shRNA) knockdown and clustered regularly interspaced short palindromic repeats (CRISPR)/CRISPR-associated protein 9 (Cas9) knockout, we identified FOXP1 and GATA3 as regulators of HIV latency and reactivation.

## Results

### Latency reversal in *in vitro* models of HIV latency

**Figure 1:**
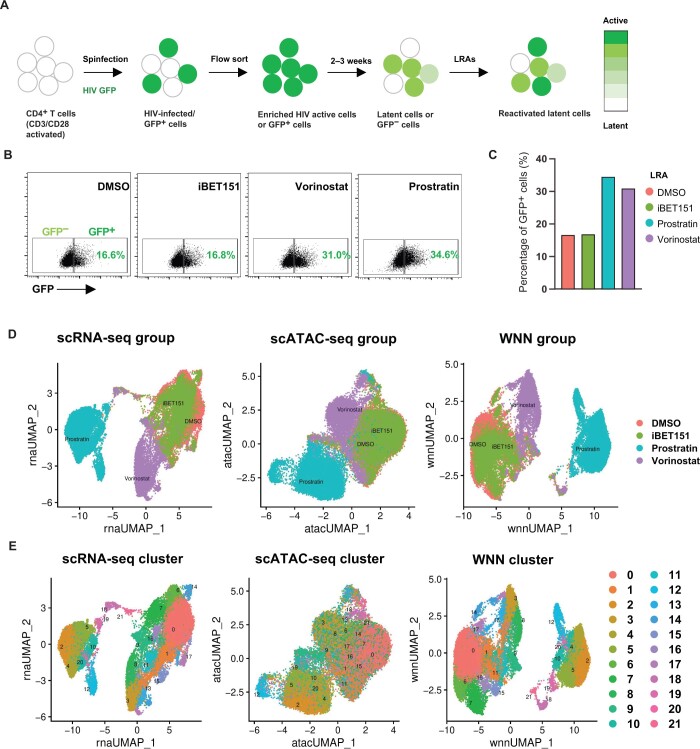
Single-cell multiomic analysis of HIV latency reversal **A**. A schematic of the experimental design of primary CD4^+^ T cell HIV latency model followed by stimulation with LRAs. DMSO was used as a control. **B**. Flow cytometry showing the expression of the viral GFP reporter in HIV-GFP-infected primary CD4^+^ T cells from donor 1 after exposure to LRAs (vorinostat, prostratin, and iBET151) or control vehicle (DMSO). **C**. Bar plot showing the percentage of GFP^+^ cells in infected cells for each condition. **D**. and** E**. UMAP dimension reduction of scRNA-seq and scATAC-seq, and WNN with cells labeled by conditions (D) and by graph-based clusters (E). Data shown here are from primary CD4^+^ T cells of donor 1. Data from 2D10 cells and primary CD4^+^ T cells of donor 2 are shown in Figures S1 and S2, respectively. LRA, latency reversing agent; HIV, human immunodeficiency virus; GFP, green fluorescent protein; DMSO, dimethylsulfoxide; WNN, weighted nearest neighbor; UMAP, Uniform Manifold Approximation and Projection; scRNA-seq, single-cell RNA sequencing; scATAC-seq, single-cell assay for transposase-accessible chromatin with sequencing.

To understand the relationship between cellular transcriptome, chromatin state, and HIV transcription, we stimulated 2D10 cells [24] — a Jurkat cell line model of HIV latency, and *in vitro* latently infected primary CD4^+^ T cells from two different donors with one of three different LRAs, or with control vehicle [dimethylsulfoxide (DMSO)] (**Figure 1**A, Figures S1A and S2A). The primary CD4^+^ T cell model of latency has been previously described [31] and involves infection of activated CD4^+^ T cells with a reporter strain of HIV encoding a destabilized enhanced green fluorescent protein (HIV-GFP), followed by culture for 3 weeks to allow loss of viral gene expression as the cells return to a resting state (Figure 1A, Figure S2A). The LRAs that we used represent different mechanisms of action — histone deacetylase inhibitor (HDACi) by vorinostat, bromodomain inhibitor (BRDi) by iBET151, and protein kinase C activator (PKCa) by prostratin. For 2D10 cells, we added limiting amounts of each LRA to induce HIV reactivation in only a subset of cells (up to 20%–30%) at 24 h post stimulation (Figure S1B). Approximately 7% of the 2D10 cells displayed some level of background HIV expression as measured by detection of GFP in the absence of LRA stimulation. For the HIV-GFP infected primary CD4^+^ T cells, a GFP^+^ population of 15%–20% was observed in the absence of LRA stimulation (Figure 1B and C, Figure S2B and C). Notably, while vorinostat and prostratin both significantly increased the fraction of GFP^+^ primary CD4^+^ T cells for both donors by 10%–15%, iBET151 had no apparent latency reversing effect in either primary cell donor, indicating differences between the cell line model and primary CD4^+^ T cells (Figure 1C, Figures S1B and S2C).

Each population was then analyzed by integrated scRNA-seq/scATAC-seq, yielding data for a total of ∼ 4000 cells for 2D10 cells, ∼ 80,000 cells for primary CD4^+^ T cells from donor 1, and ∼ 40,000 cells for primary CD4^+^ T cells from donor 2. Quality control metrics indicated high-quality library preparation and sequencing quality with well-balanced depth of coverage between the assay for transposase-accessible chromatin (ATAC) and RNA domains (Figure S3). For both 2D10 cells and primary CD4^+^ T cells, dimension reduction using Uniform Manifold Approximation and Projection (UMAP) [32] of the scRNA-seq data showed a clear separation of vorinostat- and prostratin-treated cells from the DMSO-treated (control) cells along UMAP_1 and UMAP_2, indicating LRA-specific modulation of cellular genes (Figure 1D, Figures S1C and S2D). iBET151-stimulated primary CD4^+^ T cells, by contrast, mostly overlapped with DMSO-treated cells, indicating a limited impact of this drug on the cellular transcriptome, consistent with our observation of limited effect on viral transcription at this concentration. For primary CD4^+^ T cells, UMAP display of the matching scATAC-seq data also indicated separation of prostratin- and vorinostat-treated cells from DMSO- and iBET151-treated cells (Figure 1D, Figure S2D). For 2D10 cells, separation of the four conditions at the epigenomic level was less clear — DMSO- and iBET151-treated cells occupied overlapping regions of the scATAC-seq UMAP plot, while vorinostat- and prostratin-treated cells were partially separated from other cell conditions (Figure S1C).

We also labeled clusters of cells based on their transcriptomes, irrespective of condition, using graph-based clustering. We observed 10 clusters for 2D10 cells, and 22 and 20 clusters for primary CD4^+^ T cell donors 1 and 2, respectively (Figure 1E, Figures S1D and S2E). Each cluster exhibited a distinct expression profile indicated by the set of RNAs that were most selectively enriched in each cluster (Table S1). This clustering pattern indicated that diverse states of the cells exist within each condition. Similar to our observations when the cells were labeled by condition, we observed that these transcriptomic clusters were still observable but somewhat harder to discern from the scATAC-seq data, particularly for 2D10 cells. Overall, we observed that clusters that were adjacent in the scRNA-seq UMAP plots were typically adjacent in scATAC-seq UMAP plots as well.

### Heterogeneous viral gene expression in individual cells

We next examined the expression of HIV transcripts across the cell populations. Reads were aligned to a HIV reference genome derived from the viral clones used to generate the 2D10 cells and used to infect the primary CD4^+^ T cells [33]. For 2D10 cells, the majority of cells (80%) did not express detectable vRNAs in the absence of LRA stimulation. All three LRA stimulations caused a significant increase in the abundance of HIV mapping reads in 2D10 cells, with significant *P* values from the Tukey test and one-way analysis of variance (ANOVA) test (Figure S4A). Specifically, 20% of cells had detectable vRNAs with at least one unique molecular identifier (UMI) read for DMSO-treated cells, whereas the percentages were 41%, 52%, and 43% upon treatments with iBET151, prostratin, and vorinostat, respectively. The higher fraction of 2D10 cells with detectable vRNAs compared to the GFP^+^ fraction observed by flow cytometry (Figure S1B) indicates that flow cytometry for GFP underestimates the fraction of cells with active viral transcription. Abundance of vRNA reads across the vRNA-expressing cells within each condition was highly variable and ranged from 0.6% to 17.3% of the reads for each cell (Figure S4B).

**Figure 2:**
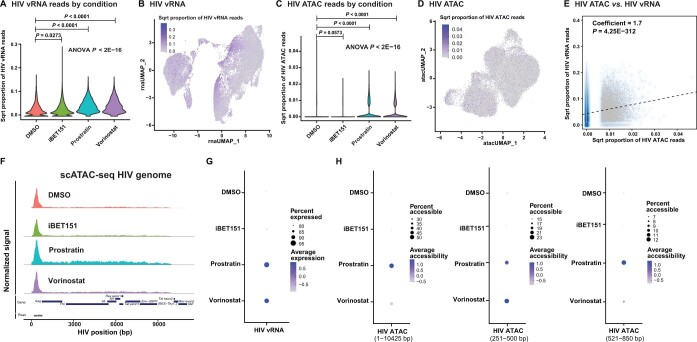
HIV vRNA expression and chromatin accessibility **A**. Transformed HIV vRNA reads under different treatment conditions. *P* values from one-way ANOVA test and for significant pairwise differences based on the Tukey test are shown. **B**. UMAP plot of the scRNA-seq data color-coded based on the level of HIV vRNA expression. **C**. Transformed HIV mapping scATAC-seq reads under different treatment conditions. *P* values from one-way ANOVA test and for significant pairwise differences based on the Tukey test are shown. **D**. UMAP plot of the scATAC-seq data color-coded based on the levels of HIV mapping scATAC-seq reads. **E**. Correlation scatter plot showing the sqrt proportion of scATAC-seq reads mapping to HIV (X-axis) *vs.* the sqrt proportion of scRNA-seq reads mapping to HIV (Y-axis) across the cell population. **F**. Coverage plots showing normalized read counts from scATAC-seq along the HIV reference genome for different LRA conditions. The dark blue and gray boxes represent gene annotations and ATAC-seq peaks, respectively. **G**. Dot plot showing the percentage of cells expressing HIV and the average expression level for vRNAs. **H**. Percentage of cells with accessible HIV DNA and the average accessibility for different regions of the HIV proviral genome. Data shown here are from primary CD4^+^ T cells of donor 1. Data from 2D10 cells and primary CD4^+^ T cells of donor 2 are shown in Figures S4 and S5, respectively. ANOVA, analysis of variance; ATAC, assay for transposase-accessible chromatin; scATAC-seq, single-cell assay for transposase-accessible chromatin with sequencing; vRNA, viral RNA; RNA-seq, RNA sequencing; sqrt, square root.

For primary CD4^+^ T cells, we observed that, for both donors, the majority of infected cells (∼ 80%) exhibited a low but detectable vRNAs even in the absence of LRA stimulation, even though only ∼ 15%–20% of cells had detectable GFP expression by flow cytometry, indicating that most infected cells in this model retain low levels of persistent viral transcription (**Figure 2**A and B, Figure S5A and B). Nevertheless, both prostratin and vorinostat caused a significant up-regulation of vRNA abundance across the primary CD4^+^ T cell populations, indicating transcriptional up-regulation, although prostratin was less potent at up-regulating vRNAs in donor 2 than in donor 1 (Figure 2A, Figure S5A). By contrast, iBET151 caused only a small increase in vRNA abundance in both primary CD4^+^ T cell donors, consistent with the limited transcriptomic and epigenomic impact of this compound in primary CD4^+^ T cells (Figure 2A, Figure S5A). The reason for the difference in potency of iBET151 between 2D10 cells and primary CD4^+^ T cells is unclear and may reflect differing roles of the bromodomain (BRD)-containing targets of iBET151 in enforcing latency between cell lines and primary CD4^+^ T cells.

We next examined whether induction of viral gene expression was linked to the transcriptomic or epigenomic characteristics of the infected cells. UMAP display of the scRNA-seq data showed that vRNA-expressing cells were enriched in specific regions of the plots/clusters (Figure 2B, Figures S4C, S4D, and S5B). This clustering of vRNA-expressing cells was less prominent when vRNAs were excluded from the dataset when performing clustering, indicating that the viral transcripts themselves are contributing to the formation of these clusters (Figures S6–S8). Nevertheless, vRNA-expressing cells displayed a non-random distribution across the scRNA-seq UMAP plot even without inclusion of vRNAs, indicating that reactivation of viral gene expression is correlated with transcriptional profiles within the host cells.

### Viral gene expression is correlated with increased accessibility of the viral genome

To understand how the chromatin configuration of the viral genome was related to viral genome expression, we next examined the accessibility of the HIV provirus within our datasets by examining scATAC-seq reads that mapped to the HIV reference genome. This approach has been previously used to identify HIV-infected cells in samples from people with HIV [34]. Overall, the HIV mapping scATAC-seq data for both model systems were sparse, with the majority of cells exhibiting no detectable HIV mapping scATAC-seq reads (Figure 2C and D, Figure S5C and D). Notably, we observed statistically significant (*P* = 4.25E−312 for donor 1 and *P* = 0 for donor 2) correlation between HIV mapping scATAC-seq reads and the abundance of vRNAs within each cell for both primary CD4^+^ T cell donors (Figure 2E, Figure S5E), consistent with the hypothesis that viral gene expression is linked to overall proviral chromatin accessibility. This correlation between viral gene expression and proviral chromatin accessibility is further recapitulated in the 2D10 cells (Figure S4E–G). When we examined the proportion of scATAC-seq reads that were mapped to HIV across different stimulation conditions, we observed differential impacts of the LRAs on proviral accessibility. Specifically, vorinostat caused increased overall proviral accessibility in both primary CD4^+^ T cell donors, while prostratin increased proviral accessibility in donor 1, but led to a more modest change in donor 2, consistent with its lower impact on vRNA abundance in this donor (Figure 2C and F–H, Figure S5C, F, and G). iBET151 had little impact on proviral accessibility in either primary CD4^+^ T cell donor, also consistent with its lack of latency reversing activity in this system (Figure 2C, Figure S5C).

By aggregating scATAC-seq data for all cells within each condition and examining read density across the viral genome, we observed distinct accessibility peaks within the HIV genome for 2D10 cells and for primary CD4^+^ T cells (Figure 2F, Figures S4E and S5F). These peaks were located at the 5′ end of the viral genome, overlapping with the 5′ long terminal repeat (LTR). The HIV genome contains several known nucleosomal positions within the 5′ end of the virus (Nuc0, Nuc1, Nuc2, and Nuc3) [35–38]. The most prominent peak (position: 251–550 bp) for both 2D10 cells and primary CD4^+^ T cells corresponds to a known DNase I hypersensitive (DHS) region between Nuc0 and Nuc1 and overlaps the viral transcription start site (TSS) at position 455 bp. In 2D10 cells, a second prominent peak was observed immediately downstream of Nuc1 between Nuc1 and Nuc2 (position: 521–850 bp) (Figure S4E). In primary CD4^+^ T cells, the LTR promoter associated peak displayed increased accessibility in response to vorinostat and prostratin in donor 1, and in response to vorinostat in donor 2, indicating that, in primary CD4^+^ T cells, increased promoter accessibility is a key aspect of potent latency reversal. We observed a consistent increase in accessibility across the gene body of the HIV provirus in prostratin-treated cells for both primary CD4^+^ T cell donors, highlighting that this LRA is particularly effective at promoting increases in accessibility downstream of the promoter associated peaks (Figure 2F–H, Figure S5F and G).

Overall, these observations are consistent with the hypothesis that an overall increase in accessibility across the HIV genome is correlated with increased viral gene expression, likely due to nucleosomal remodeling and histone modification to permit passage of RNA Pol II through the provirus. These data also indicate significant differences between LRAs in terms of the impact on accessibility and transcription during viral reactivation.

### LRAs promote defined changes to the transcriptome and epigenome of HIV-infected cells

**Figure 3:**
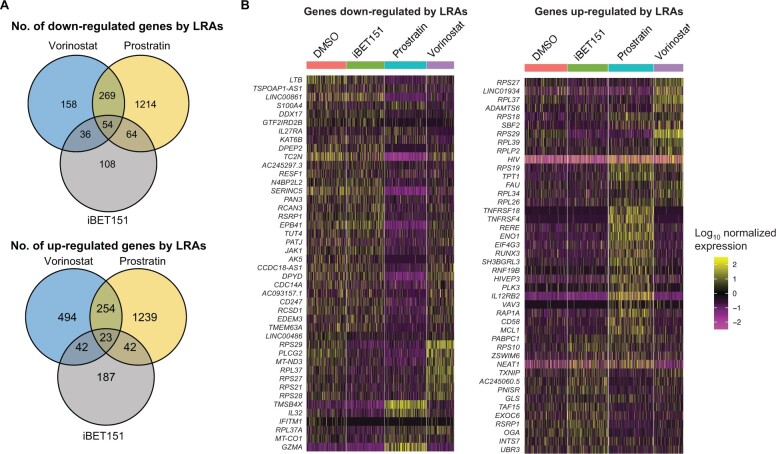
DEGs stimulated by LRAs **A**. Venn diagrams showing the number of down-regulated (upper) and up-regulated (lower) genes upon treatment with LRAs (FDR-adjusted *P* value < 0.05). **B**. Heatmaps showing normalized and scaled RNA expression levels for different treatment conditions. The rows contain representative genes that were down-regulated (left) and up-regulated (right), respectively. The columns represent single cells. Data shown here are from primary CD4^+^ T cells of donor 1. Data from 2D10 cells and primary CD4^+^ T cells of donor 2 are shown in Figures S9 and S10, respectively. FDR, false discovery rate.

To understand the impact of individual LRAs on host cells, we examined the host-cell transcripts that were up-regulated or down-regulated by the addition of the LRAs. As expected, each LRA displayed a distinct transcriptomic signature when considering the up-regulated genes (**Figure 3**, Figures S9 and S10). In general, prostratin had the largest impact on the transcriptome [1558 differentially expressed genes (DEGs) in donor 1], consistent with its known ability to activate multiple signaling pathways, while vorinostat (813 DEGs in donor 1) and iBET151(294 DEGs in donor 1) had more modest impacts. In 2D10 cells, only eight genes/transcripts exhibited increased expression with all three LRAs (Figure S9A), while in primary CD4^+^ T cells, 23 and 7 up-regulated transcripts were shared by the three LRAs for donors 1 and 2, respectively (Figure 3A, Figure S10A).

To probe the biological significance of the DEGs, we performed Gene Ontology (GO) enrichment analysis [39] to identify enrichment of specific functional classes of genes. For the down-regulated genes in 2D10 cells, we detected a shared set of enriched biological process terms across all three LRAs (Table S2). For the down-regulated genes in primary CD4^+^ T cells, some of the enriched sets include T cell activation (GO:0042110) and host–virus interaction (KW-0945) (Tables S3 and S4). For the up-regulated genes, the GO enrichment analyses in 2D10 cells and primary CD4^+^ T cells indicated that prostratin induced transcriptional responses consistent with the activation of T cell receptor, tumor necrosis factor (TNF), Rap-1, PD-1 checkpoint, FOXO/MAPK/RAS, and NF-κB signaling pathways. Inhibition of T cell, NF-κB, and MAPK/RAS signaling pathways block reversal of HIV expression, suggesting that prostratin treatment provides sufficient signals for HIV expression in latently infected cells. Conversely, we did find T cell receptor signaling pathways as common figure between 2D10 cells and primary CD4^+^ T cells induced by vorinostat (Tables S2–S4).

**Figure 4:**
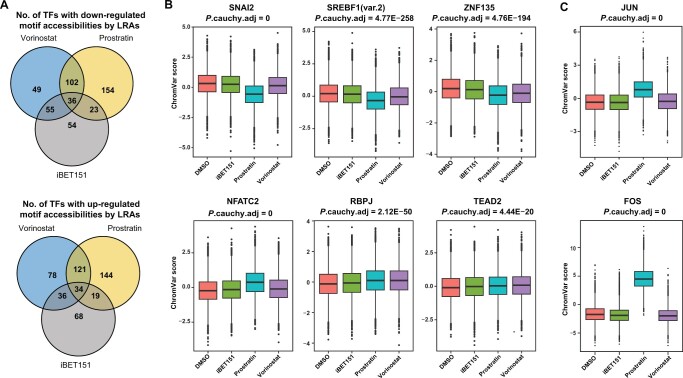
DATFs stimulated by LRAs **A**. Venn diagrams showing the number of TFs whose motif accessibilities were significantly down-regulated (upper) or up-regulated (lower) upon treatment with LRAs compared with DMSO (FDR-adjusted *P* value < 0.05). **B**. Box plots showing distributions of motif accessibilities (deviation scores) of selected TFs with decreased accessibility scores (SNAI2, SREBF1, and ZNF135; upper) or increased accessibility scores (NFATC2, RBPJ, and TEAD2; lower) in response to all three LRAs. *P* values from Wilcoxon rank sum tests comparing DMSO-treated and one of the LRA-treated samples were integrated using the Cauchy combination. **C**. Box plots showing distributions of motif accessibilities of positive controls, FOS and JUN. Data shown here are from primary CD4^+^ T cells of donor 1. Data from primary CD4^+^ T cells of donor 2 and 2D10 cells are shown in Figures S11 and S12, respectively. TF, transcription factor; DATF, differentially accessible transcription factor.

We next examined the impact of the LRAs on the cellular chromatin of HIV-infected cells by using the scATAC-seq data to examine enrichment of TF-binding sites within the open chromatin. During activation of a TF in a cell, TF binding to promoter and enhancer regions is frequently associated with an increase in accessibility at binding sites due to the action of nucleosomal remodeling complexes recruited by the TFs. Thus, increased enrichment of binding sites for a TF in the open chromatin of a cell can serve as a proxy for overall activity of a TF in that cell. Using chromVAR [40], we calculated a TF deviation score for a set of 633 TFs with annotated binding motifs for each cell and examined the impact of each LRA on these TF deviation scores via a test of differential motif accessibility. Similar to the scRNA-seq data, each LRA caused a distinctive impact on the pattern of TF-binding motif accessibilities in the host cells (**Figure 4**, Figures S11 and S12). Each LRA modulated the motif accessibilities of a set of TFs that were unique to each LRA, but there were also a set of TFs whose motif accessibilities were coregulated by all three LRAs. Consistent with its more potent transcriptomic impact, prostratin up-regulated the motif accessibilities of the greatest number of TFs in both 2D10 cells and primary CD4^+^ T cells. Vorinostat and iBET151 had milder impact on the host-cell TF deviation scores in primary CD4^+^ T cells, in concordance with the differential gene expression analysis. Notably, TF deviation scores were significantly increased after prostratin stimulation for two members of the AP-1 family (JUN and FOS), consistent with this family’s known role in the PKC signaling pathway (Figure 4C, Figure S11C). In 2D10 cells, no TFs displayed increased motif accessibilities by all three LRAs; however, four TFs exhibited down-regulated motif accessibilities in response to all three LRAs, including FIGLA (a bHLH TF) as well as SNAI1, SNAI2, and SNAI3 (three members of the SNAIL family of transcriptional repressors) (Figure S12C). In primary CD4^+^ T cells, 34 and 24 TFs displayed increased motif accessibilities by all three LRAs in donors 1 and 2, respectively, while the motif accessibilities of 36 and 28 TFs were down-regulated by all three LRAs for donors 1 and 2, respectively (Figure 4A, Figure S11A). Seven of the TFs with up-regulated motif accessibilities and 11 of the TFs with down-regulated motif accessibilities overlapped between primary CD4^+^ T cell donors. This down-regulated set also included members of the SNAIL family, indicating a conserved pathway between cell lines and primary CD4^+^ T cells. Additionally, the regulatory roles of these differentially accessible TFs (DATFs) were corroborated by the enrichment of Tn5 integration events in the flanking regions of the TF-binding sites using DNA footprinting analyses (Figure S13).

### Identification of cellular transcripts that correlate with HIV transcription

**Figure 5:**
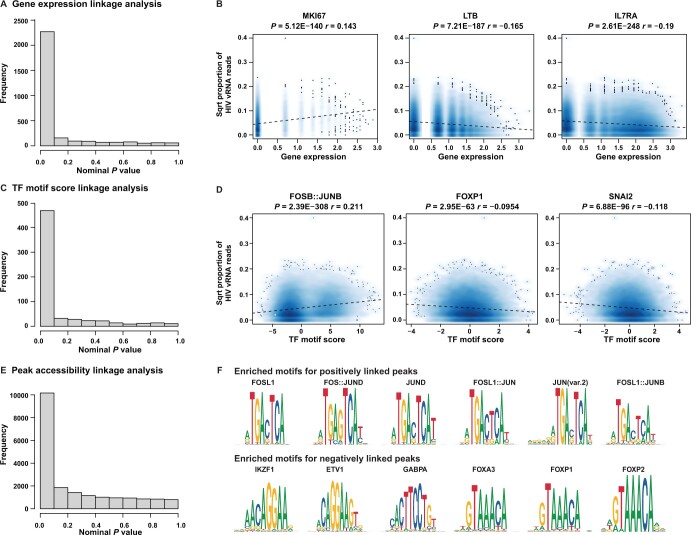
Linkage analyses between HIV transcription and cellular transcripts/TFs/peaks **A**. Distribution of nominal *P* values from cellular RNA expression linkage analysis. **B**. Visualization of selected cellular transcripts linked with HIV vRNA expression. The dotted line represents the fitted line from a simple regression. Spearman correlation coefficient (*r*) and nominal *P* value are shown. **C**. Distribution of nominal *P* values from TF activity linkage analysis. **D**. Visualization of activities of selected TFs linked with HIV vRNA expression. **E**. Distribution of nominal *P* values from peak linkage analysis. **F**. Visualization of motifs enriched in ATAC-seq peaks that were positively (top) and negatively (bottom) associated with the HIV vRNA expression. Data shown here are from primary CD4^+^ T cells of donor 1. Data from 2D10 cells and primary CD4^+^ T cells of donor 2 are shown in Figures S14 and S15, respectively.

To understand the relationship between vRNA, cellular RNA, and chromatin accessibility more deeply, we examined pairwise correlations between summed vRNA levels for each cell and individual cellular transcript levels across cells of the dataset (**Figure 5**A, Figures S14A and S15A). We performed this analysis for each individual condition, as well as across the aggregated cell population. Although we found some condition-specific associations, we observed that including all conditions in this analysis identified a significantly greater number of HIV-correlated transcripts, likely due to the increased cellular diversity and statistical power provided by the larger cell numbers (Figures S16–S18). Therefore, we focused our analysis on the aggregated cell populations. In 2D10 cells, we identified 229 and 123 cellular transcripts whose expression was correlated positively or negatively with HIV vRNA levels using a 0.05 cutoff of false discovery rate (FDR)-adjusted *P* value, respectively (Tables S5A and S6). In primary CD4^+^ T cells, we observed 2437 (donor 1) and 1204 (donor 2) transcripts that were significantly correlated with HIV vRNA levels. The top 20 significantly linked genes in primary CD4^+^ T cells are included in **Table 1** and Table S7A for donors 1 and 2, respectively, with the full testing results shown in Tables S8 and S9.

**Table 1:** Top 20 genes significantly linked with HIV transcription in primary CD4^+^ T cells of donor 1

Gene	Spearman correlation coefficient	Nominal *P* value	FDR-adjusted *P* value
*TC2N*	–0.2281	9.41E–321	2.70E–318
*LINC00861*	–0.2056	1.69E–251	1.39E–249
*LTB*	–0.1815	8.07E–196	3.31E–194
*CENPF*	0.1242	4.03E–169	9.27E–168
*BACH2*	0.1574	7.30E–147	1.20E–145
*MKI67*	0.1294	1.22E–138	1.63E–137
*CD69*	0.1634	7.59E–128	8.08E–127
*SLC41A2*	0.1242	1.63E–107	1.14E–106
*CD109*	0.1246	8.00E–99	4.46E–98
*KIF3B*	0.1292	6.19E–97	3.30E–96
*CASK*	–0.1181	5.48E–85	2.40E–84
*HDAC7*	0.1357	2.92E–84	1.25E–83
*LMNA*	0.1243	2.46E–77	9.68E–77
*MYO1E*	0.1022	3.19E–73	1.12E–72
*IL2RB*	0.1036	1.97E–65	5.82E–65
*FRMD4B*	0.1064	4.02E–61	1.07E–60
*IL32*	0.0963	1.35E–51	2.97E–51
*IL17RA*	–0.0882	1.30E–49	2.77E–49
*STRBP*	0.1040	5.42E–46	1.06E–45
*BCL2L1*	0.0825	7.39E–43	1.35E–42

*Note*: HIV, human immunodeficiency virus; FDR, false discovery rate.

Overall, each transcript in these sets individually correlated with HIV vRNA levels only weakly, albeit the small *P* values, with a 95% confidence interval for the significant $R^{2}$. The most highly correlated cellular transcript in 2D10 cells was *TSPOAP1* (Figure S14B), which encodes a cytoplasmic protein with no previously known role in HIV replication. Although the cellular role of TSPOAP1 is unclear, it has recently been shown that TSPOAP1 enhances influenza A virus replication by antagonizing innate immune signaling [41]. Interestingly, two of the most significantly correlated transcripts in 2D10 cells were long non-coding RNAs, *MALAT1* and *NEAT* (Figure S14B), both of which have been reported to be implicated in HIV replication [42].

Amongst the highly HIV-correlated transcripts in primary CD4^+^ T cells, several noteworthy genes were observed. The transcript for the centromere protein CENPF was significantly positively associated with HIV transcription in both donors. Additionally, transcripts for two proteins associated with active cell metabolism and cell division (GAPDH and MKI67) were significantly correlated with vRNA levels. The transcriptional regulator BACH2, which has been previously identified as a recurrent integration target for HIV, was also highly associated with HIV transcription [5]. Overall, there was a pattern of increased expression of T cell activation markers with greater HIV vRNA levels — specifically, expression of transcripts for the activation markers CD38, IL2RA (CD25), and CD69 was positively correlated with vRNA levels in both donors. Similar to 2D10 cells, *MALAT1* expression was positively correlated with vRNA levels in donor 2 but was uncorrelated in donor 1. Interestingly, the transcript for the DNA helicase TOP2A, which is required for unwinding of DNA templates during transcription, was also positively correlated with HIV transcription [43].

When we examined genes whose transcription was negatively correlated with vRNA levels in primary CD4^+^ T cells, we observed that high expression of transcripts for LTB and IL7RA was associated with lower vRNA levels (Figure 5B, Figure S15B). These two genes are highly expressed in quiescent memory T cells, consistent with our previous observations that HIV latency preferentially occurs in cells with a quiescent memory phenotype [31]. Notably, expression of the transcripts encoding two well characterized HIV restriction factors, SERINC5 and SAMHD1, was negatively correlated with HIV transcription across the cell population. Expression of *STK38*, encoding a kinase that has been shown to be incorporated into HIV particles, was also negatively correlated with HIV vRNA levels, along with numerous genes with no known previous roles in HIV infection, such as *TC2N* and the long intergenic non-protein coding RNA *LINC00861*.

We further carried out a GO enrichment analysis using a set of genes that are significantly linked with vRNA levels. In 2D10 cells, we observed that correlated transcripts were enriched in the GO terms such as translation, cytoplasmic translation, and ATP binding (Table S10); in primary CD4^+^ T cells, enrichment was observed in the GO terms such as DNA binding, ATP binding, metal ion binding, and T cell receptor signaling pathway (Table S10).

We then focused on TFs whose transcripts were found to be correlated with vRNA levels. In 2D10 cells, *GATA3* was the most significantly positively correlated TF-coding transcript (*P* = 0.0005), and interestingly, *CTCF* was the most significant negatively associated TF-coding transcript (*P* = 0.0072). We have recently reported that CTCF is a novel repressor of HIV gene expression in CD4^+^ T cells, and knockdown of *CTCF* expression in latently infected Jurkat cells and primary CD4^+^ T cells reactivates HIV gene expression [44]. Transcripts for YY1 and HSF1, two TFs that have been confirmed to repress HIV replication, were also found to be negatively correlated with vRNA levels in 2D10 cells. In primary CD4^+^ T cells, we found that transcripts encoding the BACH2, ETV6, and IRF4 TFs were among the top positively correlated TFs for both donors. Members of the AP-1, NF-κB, and RUNX families of TFs were also highly represented within the set of positively correlated factors, while the transcripts of the TCF, KLF, and forkhead (FOX) TFs were negatively correlated with vRNA levels. Overall, these data indicate that this approach not only successfully identifies known regulators of HIV expression and reactivation, but also reveals novel HIV associations with cellular transcripts that could represent important but uncharacterized viral regulators.

### Activities of individual TFs correlate with HIV transcription

The integrated multiomic approach also allowed us to examine connections between the epigenomic state of the cells and HIV transcription. Using the chromVAR motif scores calculated from the scATAC-seq reads for each cell, we investigated whether activity of individual TFs correlates with vRNA levels across the cell population. We expect that TFs that promote HIV transcription will have motif deviation scores that positively correlate with vRNA levels, while TFs that repress HIV will have motif deviation scores that negatively correlate with vRNA levels. The top 20 significantly linked TFs for primary CD4^+^ T cells of donor 1 and donor 2 are included in **Table 2** and Table S7B, respectively. In 2D10 cells, 31 TFs had motif deviation scores that were positively or negatively correlated with vRNA levels using a cutoff of FDR-adjusted *P* value 0.05 (Figure S14C and D; Table S5B). Similar to the correlations between the vRNA level and the abundance of cellular transcripts, the overall Spearman correlation coefficients between vRNA levels and TF deviation scores, although statistically significant, were individually weak (95% confidence interval for the $R^{2}$). Among TFs positively correlated with HIV vRNA levels in 2D10 cells, multiple members of the ETS, GATA, and AP-1 families were identified. Notably, the HIV LTR promoter contains binding sites for members of all three of these families, and previous work has shown that individual members of these families can positively regulate HIV gene expression, indicating that this approach successfully identifies HIV regulators [45–49]. Amongst the negatively correlated TFs in 2D10 cells, two families of TFs were prominently represented — TCF and SNAIL families. In particular, three members of the TCF family were associated with lower HIV vRNA levels — TCF3, TCF4, and TCF12. Some previous data have linked a related TCF protein (TCF1) to HIV regulation through direct binding [50]. Additionally, TCF4 has been shown to repress HIV transcription [51,52]. Interestingly, our analysis also identified SNAI1, SNAI2, and SNAI3 (members of the SNAIL family of transcriptional repressors) as being negatively correlated with HIV transcription. This family of TFs binds to E-box motifs and has not been previously implicated in HIV transcription.

**Table 2:** Top 20 TFs significantly linked with HIV transcription in primary CD4^+^ T cells of donor 1

TF	Motif	Spearman correlation coefficient	Nominal *P* value	FDR-adjusted *P* value
FOSB::JUNB	MA1135.1	0.2047	2.39E–308	6.24E–307
FOSL2::JUNB	MA1138.1	0.2042	2.59E–308	6.24E–307
FOS::JUND	MA1141.1	0.2017	3.26E–306	3.62E–305
FOSL2::JUND	MA1144.1	0.2044	3.31E–306	3.62E–305
FOSL1::JUN	MA1128.1	0.2010	4.54E–306	4.20E–305
FOS::JUNB	MA1134.1	0.2020	2.29E–304	1.56E–303
BACH2	MA1101.2	0.2081	4.09E–302	2.46E–301
FOSL2	MA0478.1	0.2020	8.63E–300	4.95E–299
NFE2	MA0841.1	0.2044	5.96E–295	3.12E–294
NFE2L1	MA0089.2	0.1825	9.54E–234	4.60E–233
CREB1	MA0018.4	0.1505	1.39E–136	6.22E–136
FOSL2::JUNB(var.2)	MA1139.1	0.1463	1.22E–124	5.23E–124
RELA	MA0107.1	0.1352	3.39E–118	1.36E–117
JDP2(var.2)	MA0656.1	0.1417	8.95E–118	3.48E–117
FOSR::JUN	MA1127.1	0.1387	4.58E–115	1.58E–114
REL	MA0101.1	0.1329	1.09E–109	3.66E–109
FOSL2::JUND(var.2)	MA1145.1	0.1357	8.57E–109	2.79E–108
FOS::JUN(var.2)	MA1126.1	0.1201	1.08E–85	2.95E–85
SREBF1(var.2)	MA0829.2	–0.0844	1.36E–42	2.00E–42
STAT1	MA0137.3	0.0709	2.44E–31	3.03E–31

*Note*: TF, transcription factor.

In primary CD4^+^ T cells, we identified 174 TFs whose motif deviation scores were significantly correlated with vRNA levels in both donors (Figure 5C and D, Figure S15D and E). For both donors, members of the AP-1 and NF-κB (RelA, RelB) families of TFs were highly represented amongst the most positively correlated TFs, consistent with the known roles of these factors in activation of HIV transcription. Among the negatively correlated TFs, we observed transcripts for several members of the FOX TF family represented for both donors, including FOXP1, FOXO3, FOXO4, and FOXO6. This finding is consistent with our previous observations from bulk ATAC-seq of HIV-infected primary CD4^+^ T cells that transcript levels for FOX TFs are also negatively associated with HIV vRNA levels [44]. Similar to 2D10 cells, transcripts of members of the SNAIL family of transcriptional repressors, as well as those of members of the TCF family, were also negatively associated with vRNA levels (Tables S8 and S9).

### Dynamic chromatin peaks that correlate with HIV transcription are enriched for binding sites of specific TFs

We also examined the correlation between accessibility at individual cellular chromatin peaks and HIV transcription. In general, in 2D10 cells, correlations between individual peaks and HIV transcription were even weaker than those for transcripts and for TF deviation scores, with typical Spearman correlation coefficients between 0.02 and 0.05 (Figure S14E and F; Table S5C). Note that the statistical power is low due to the large number of testing/peaks and the data sparsity. Nevertheless, in 2D10 cells, we detected 224 peaks whose accessibility was significantly correlated with HIV vRNA levels, with a significance threshold of 0.01 on the nominal *P* values. In primary CD4^+^ T cells, we found 6379 and 1886 peaks that were correlated with vRNA levels in donor 1 and donor 2, respectively (Figure 5E, Figure S15G).

We then examined these sets of positively and negatively linked peaks for motif enrichment compared with a set of background peaks using Signac [53]. In 2D10 cells, we found significant enrichment of motifs that represent binding sites of 91 TFs (Table S5D). Members of the specificity protein (Sp) and Kruppel-like factor (KLF) families were prominently represented in this list, including Sp4, Sp1, Sp9, and KLF15, as well as EGR1 and ZNF148 (Figure S14F). In primary CD4^+^ T cells, we found that, for both donors, the most enriched TF-binding motifs within enriched peaks were members of the AP-1, FOX, and KLF families, although enrichment of several other TF families could also be observed (Sp1, NF-κB, and ETS) (Figure 5F, Figure S15H; **Table 3**, Table S7C). These findings highlight the presence of conserved associations between cell line models of latency and primary CD4^+^ T cell models, but also indicate some important differences.

**Table 3:** Top 20 enriched TF-binding motifs significantly linked with HIV transcription in primary CD4^+^ T cells of donor 1

TF	Motif	Observed	Background	Percent.observed	Percent.background	Fold enrichment	*P* value	FDR-adjusted *P* value
FOS::JUND	MA1141.1	1359	2055	15.57%	9.43%	1.65	3.81E–139	1.20E–136
FOSL1::JUNB	MA1137.1	1281	1906	14.68%	8.74%	1.68	5.68E–139	1.20E–136
FOS::JUNB	MA1134.1	1185	1734	13.58%	7.95%	1.71	3.83E–136	6.05E–134
FOSL1::JUN	MA1128.1	1359	2069	15.57%	9.49%	1.64	1.92E–135	2.44E–133
JUND	MA0491.2	1332	2021	15.26%	9.27%	1.65	2.54E–134	2.68E–132
FOSL1::JUND	MA1142.1	1346	2063	15.42%	9.46%	1.63	1.83E–130	1.66E–128
FOSL2::JUN	MA1130.1	1279	1934	14.66%	8.87%	1.65	2.87E–130	2.27E–128
JUNB	MA0490.2	1407	2204	16.12%	10.11%	1.59	3.49E–125	2.21E–123
FOSL2::JUND	MA1144.1	1184	1780	13.57%	8.16%	1.66	1.25E–122	6.10E–121
FOS::JUN	MA0099.3	1220	1853	13.98%	8.50%	1.64	1.27E–121	5.36E–120
FOSB::JUNB	MA1135.1	1189	1810	13.62%	8.30%	1.64	3.08E–117	1.22E–115
FOSL2::JUNB	MA1138.1	1199	1861	13.74%	8.54%	1.61	2.59E–109	9.63E–108
KLF5	MA0599.1	4188	8624	47.99%	39.56%	1.21	6.53E–96	2.07E–94
JUN::JUNB	MA1132.1	1007	1556	11.54%	7.14%	1.62	1.88E–92	5.67E–91
ZNF148	MA1653.1	4281	8890	49.05%	40.78%	1.20	1.10E–91	2.90E–90
KLF4	MA0039.4	4274	8986	48.97%	41.22%	1.19	1.55E–80	3.50E–79
MAZ	MA1522.1	3451	7304	39.54%	33.50%	1.18	1.27E–53	2.01E–52
ZNF682	MA1599.1	1990	4171	22.80%	19.13%	1.19	2.87E–29	3.56E–28
ZNF263	MA0528.2	3111	6887	35.65%	31.59%	1.13	5.18E–26	5.96E–25
RREB1	MA0073.1	3175	7093	36.38%	32.54%	1.12	3.05E–23	3.33E–22

*Note*: A list of the top 20 TF-binding motifs enriched in ATAC-seq peaks that were significantly associated with HIV transcription. Corresponding TFs, the numbers of motifs occurring in the linked peaks and background peaks (Observed and Background), the percentages of linked peaks and background peaks containing the motif (Percent.observed and Percent.background), fold enrichment (Percent.observed *vs.* Percent.background), *P* values from hypergeometric tests examining the degree of enrichment, and FDR-adjusted *P* values are shown as columns.

To examine the reproducibility of these datasets across donors, we compared the sets of correlated transcripts, TF activities, and TF-binding sites enriched in correlated peaks between donor 1 and donor 2. As expected, we observed an overall higher number of correlated transcripts and TF activities for donor 1, consistent with the greater transcriptomic impact of the LRAs in this donor. When we examined the correlated features for donor 2, however, the majority of these were also detected for donor 1, suggesting a significant degree of conserved associations. Specifically, 65.9% of the correlated DEGs, 83.3% of the correlated DATFs, and 88.5% of the TF-binding sites enriched in correlated peaks for donor 2 were also detected in donor 1 (Figure S15C, F, and I).

**Figure 6:**
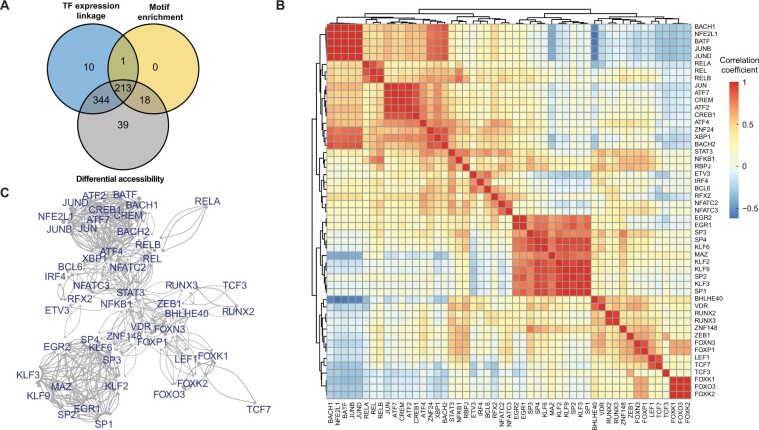
TF regulators of HIV latency reversal **A**. Venn diagram showing the number of HIV-linked TFs from three different testing schemes: expression linkage analysis of TF-coding genes (TF expression linkage), enrichment analysis of TF-binding motifs using significantly linked peaks (motif enrichment), and analysis of TFs with differential motif accessibility (differential accessibility). **B**. Heatmap showing correlations between TF pairs for selective 50 TFs with the highest combined *P* values from the three testing schemes shown in (A). The correlation is computed using the TF-specific consensus PCs, calculated using both the TF-coding gene expression level and its motif accessibility. **C**. TF regulatory network constructed from TFs’ motif deviation scores and expression levels (see Materials and methods for details). Data shown here are from primary CD4^+^ T cells of donor 1. Data from 2D10 cells and primary CD4^+^ T cells of donor 2 are shown in Figures S19 and S20, respectively. PC, principal component.

To examine coordinate behavior between HIV-associated TFs, we examined overlap between TFs detected as being associated with vRNA expression through three independent analyses (*i.e.*, TF-coding gene expression, differential accessibility, and motif enrichment in correlated peaks) (**Figure 6**A, Figures S19A and S20A). The list of TFs with enriched binding sites in significantly linked peaks was highly overlapped with the other two analyses. We observed that the majority of TFs with HIV-linked expression also exhibited differential accessibility that was correlated with HIV vRNA expression. We further examined pairwise correlations of consensus principal components (PCs) derived from both expression levels of TF-coding genes and deviation scores of TF-binding motifs, within a set of top 50 TFs that had the smallest *P* values from the Cauchy combination test. The pairwise correlations were visualized as heatmaps shown in Figure 6B and Figures S19B and S20B. An adjacency matrix was constructed for pairs with correlation coefficient > 0.5, with visualized networks shown in Figure 6C and Figures S19C and S20C (see Materials and methods for details). For both 2D10 cells and primary CD4^+^ T cells, the data indicated that these sets of TFs could be grouped into several distinct clusters. For 2D10 cells (Figure S19C), one cluster was composed mainly of KLF and Sp TFs but also included chromatin insulators CTCF and CTCFL. A second cluster was composed almost entirely of ETS TFs, while a third cluster contained just three TFs that are members of the SNAIL family of transcriptional repressors — SNAI1, SNAI2, and SNAI3. In primary CD4^+^ T cells (Figure 6C, Figure S20C), we could also observe clear groups of HIV-associated TFs with correlated activity for both donors. Sp1 and KLF TFs formed the largest cluster, followed by clusters that included AP-1 and ATF TFs. FOX, TCF, and RUNX TFs also formed a more loosely associated group of TFs. Overall, these results from TF network analysis shed light upon the regulatory relationships and synergies among the previously identified TFs.

### Machine learning modeling of HIV transcription from multiomic data

Since each transcript, TF deviation score, or peak was only weakly correlated with vRNA levels, we next examined whether considering multiple features of the dataset at once in a multivariate machine learning model could lead to a more predictive model of HIV gene expression from the dataset. Beginning with the 2D10 cell dataset, we first used Spearman correlation coefficients between individual features (transcripts, TF deviation scores, and peaks) and vRNA levels to select the 60 highest ranked features and then incorporated these features into a boosting-based machine learning model. To simplify the prediction goal of the model, we converted the data into a binary classification of vRNA+ and vRNA− cells based on whether vRNAs were detected in each cell. For machine learning training and evaluation, we split the data into five sets — four for separate training and one for testing. By examining the receiver operating characteristic (ROC) curves to evaluate model performance, the model was able to achieve an area under the curve (AUC) of 0.79 for the training set and 0.68 for the test set (Figure S21A). We were also able to determine the rank importance of individual features to the model performance (Figure S21B). Based on this ranking, the most important predictive features for 2D10 cells were the transcripts *TSPOAP1* and *MALAT1* followed by accessibility of binding sites for the ETS TF SPIB (MA0081.2).

**Figure 7:**
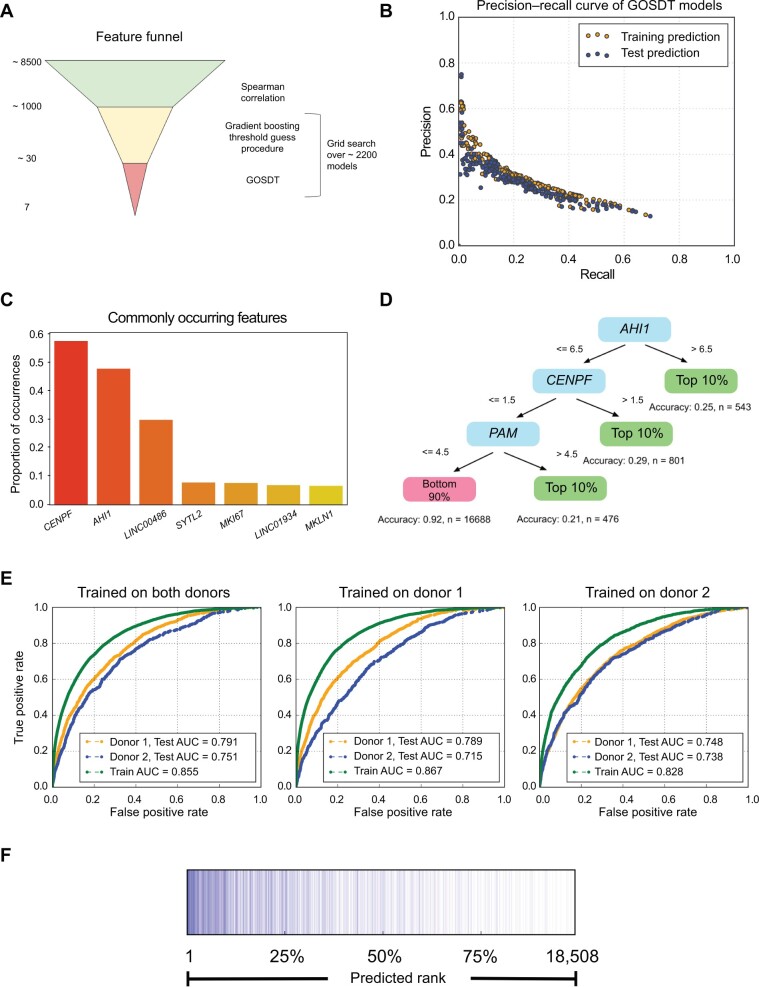
Machine learning model of HIV reactivation **A**. Schematic overview of model feature selection for GOSDT models of cells with high HIV expression (top 10%) across the dataset. Numbers to left represent the number of features used for model refinement at each stage. **B**. A set of GOSDT models trained and tested on the dataset were evaluated for precision (percentage of cells classified as high expressors that are actually high expressors) and recall (percentage of high expressors recovered) performances. Each dot represents a model. For each model, performances during training (yellow) and testing (blue) are shown. **C**. The top 7 most commonly occurring model features used by the set of GOSDT models to classify cells with respect to high HIV expression are shown as a ranked list. **D**. An example of a decision tree for predicting cells with high HIV expression (top 10%). Features that comprise this model are shown in blue, and the thresholds for data splitting are shown above each arrow. **E**. Performance of a boosting based XGBoost model predicting HIV expression levels across the cell population. The model was trained (green lines) on data from both primary CD4^+^ T cell donors (left), donor 1 only (middle), and donor 2 only (right), and tested on each donor separately — donor 1 in yellow and donor 2 in blue. AUC for each test is shown. **F**. Graphical display of the test dataset ranked by XGBoost predicted likelihood of each being within the top 10% of HIV-expressing cells (left to right), with cells that are actually within the top 10% shown as vertical blue columns. GOSDT, Generalized and Scalable Optimal Sparse Decision Trees; AUC, area under the curve.

We then applied machine learning tools to the dataset from HIV-infected primary CD4^+^ T cells. Since primary CD4^+^ T cells exhibit a more continuous level of viral gene expression, with most cells expressing some vRNAs, for these cells we focused on predicting whether a given cell was in the top 10% of vRNA expression cells based on features derived from the multiomic dataset. We first examined classification of cells using Generalized and Scalable Optimal Sparse Decision Trees (GOSDT) [54]. GOSDT is an interpretable decision tree model that generates provably optimal and sparse trees by splitting the dataset at given thresholds of individual features. To identify features of interest with respect to predicting HIV expression, the dataset was processed in three stages (**Figure 7**A). First, all features (transcript levels and TF deviation scores) were ranked by Spearman correlation coefficients against HIV expression level, and the highest-ranked 1000 features were then considered for further analysis. Second, the feature set was further reduced and binarized via the threshold guessing column elimination procedure proposed by McTavish and his colleagues [55]. A total of 2258 GOSDT models were created by varying the threshold guessing stage hyper-parameters (number of boosting stages and sample weights), and GOSDT hyper-parameters (tree depth, regularization, and sample weights controlled via random oversampling) (Figure 7B). These models were all evaluated by determining their precision (fraction of guessed cells that are actually in the top 10% of HIV-expressing cells) and their recall (the fraction of top 10% HIV-expressing cells accurately guessed). A randomly guessing model would, for example, be expected to have an AUC of the precision–recall curve of 0.1. The majority of our GOSDT models trained and tested on our data significantly outperformed this threshold, indicating significant ability to predict high HIV expression from the multiomic data. By counting the most commonly used split features in the set of final GOSDT decision trees, we observed that three cellular genes (*CENPF*, *AHI1*, and *LINC00486*) that have not been previously associated with HIV infection were the most abundantly represented features across the GOSDT models (Figure 7C and D; Table S11).

We next used a boosting-based approach to predict HIV vRNA expression across the cell population. While not as simple or interpretable as GOSDT models, boosting-derived models frequently have high predictive accuracy. An XGBoost [56] gradient boosting model was trained on the top 1000 Spearman correlated features to predict whether a given cell was within the top 10% of HIV-expressing cells. We observed that the model produced by this approach that was trained on data from both primary CD4^+^ T cell donors performed well on test sets of cells from both donors. Specifically, the AUC values were 0.791 and 0.751 for donor 1 and donor 2, respectively (Figure 7E). Additionally, the model trained on one donor still had high predictive performance for cells from the other donor, indicating biological reproducibility and robustness to inter-donor variation. When we visualized a ranked list of cells in the test set based on XGBoost prediction of being in the highest 10% of HIV-expressing cells, we observed that cells that were confirmed to be in the top 10% were highly enriched at the top of the predicted list, further demonstrating model accuracy (Figure 7F).

We also examined the importance of individual features for the performance of the XGBOOST. Interestingly, a ranked list of the most important features for this model included many highly ranked cellular genes that were also identified by the GOSDT approach, and that were also identified by our machine learning analysis of 2D10 cells, suggesting conserved pathways of importance across these models. For example, *MALAT1* and *TSMB4X* were in the top 5 features for both 2D10 cells and primary CD4^+^ T cells (Table S12).

### GATA3 and FOXP1 regulate HIV latency

**Figure 8:**
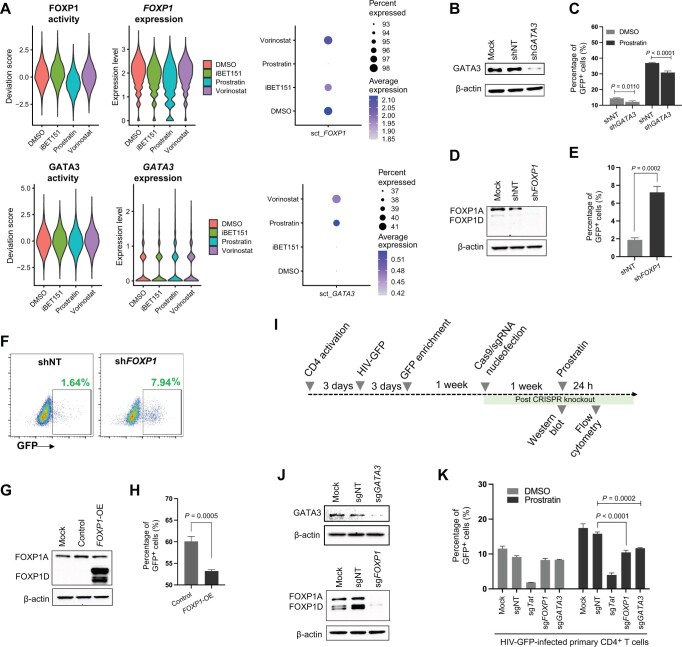
GATA3 and FOXP1 regulate HIV latency **A**. Binding site accessibility and RNA expression of GATA3 and FOXP1 across drug conditions. **B**. Western blot showing the depleted GATA3 in 2D10 cells transduced with sh*GATA3*. β-actin was used as a loading control. Non-transduced 2D10 cells were used as mock. **C**. Flow cytometry analysis of viral GFP expression in 2D10 cells transduced with sh*GATA3* or shNT at baseline and 24 h after prostratin stimulation. Data were represented by mean ± SD (*n* = 3). *P* values were determined by Student’s *t*-test. **D**. Western blot showing the depleted FOXP1 in 2D10 cells transduced with sh*FOXP1*. β-actin was used as a loading control. Non-transduced 2D10 cells were used as mock. **E**. Flow cytometry analysis of viral GFP expression in 2D10 cells at 5 days post transduction with sh*FOXP1* or shNT. Data were represented by mean ± SD (*n* = 3). *P* value was determined by Student’s *t*-test. **F**. Dot plot showing representative flow cytometry for viral GFP expression in 2D10 cells at 5 days post transduction with sh*FOXP1* or shNT. **G**. Western blot showing the overexpression of FOXP1D in HEK293T cells transfected with a FOXP1-mCherry-expressing plasmid or a mCherry-expressing plasmid (control) plus HIV-GFP. β-actin was used as a loading control. Non-transfected HEK293T cells were used as mock. **H**. Flow cytometry analysis of viral GFP expression in HEK293 cells transfected with a FOXP1-mCherry-expressing plasmid or a mCherry-expressing plasmid (control) plus HIV-GFP. Data were represented by mean ± SD (*n* = 3). *P* value was determined by Student’s *t*-test. **I**. Schematic overview of experimental design for CRISPR/Cas9 targeting of *GATA3* and *FOXP1* in HIV-GFP-infected primary CD4^+^ T cells. Activated CD4^+^ T cells were infected with HIV-GFP. At 3 days post infection, infected (GFP^+^) cells were enriched by flow sorting and cultured for 1 week before nucleofection of Cas9/sgRNA complexes targeting *GATA3* or *FOXP1*. One week post nucleofection of sgRNAs, knockout of the targets was analyzed by Western blot. Furthermore, GFP^+^ cells were quantified after stimulation with prostratin for 24 h at 1 week post nucleofection. **J**. Western blot showing the depletion of GATA3 and FOXP1 at 1 week post Cas9/sgRNA nucleofection. β-actin was used as a loading control. HIV-GFP-infected primary CD4^+^ T cells without Cas9/sgRNA nucleofection were used as mock.** K**. Flow cytometry analysis of viral GFP expression in HIV-GFP-infected primary CD4^+^ T cells nucleofected with sg*GATA3* or sg*FOXP1* at baseline and 24 h after prostratin stimulation. sgNT was used as a negative control, and sg*Tat* was used as a positive control. Data were represented by mean ± SD (*n* = 3), and *P* values were determined by one-way ANOVA Tukey’s multiple comparisons test. shRNA, short hairpin RNA; shNT, non-targeting shRNA; sh*GATA3*, *GATA3*-targeting shRNA; sh*FOXP1*, *FOXP1*-targeting shRNA; FOXP1A, FOXP1 isoform A; FOXP1D, FOXP1 isoform D; *FOXP1*-OE, *FOXP1* overexpression; sgRNA, single-guide RNA; sgNT, non-targeting sgRNA; sg*GATA3*, *GATA3*-targeting sgRNA; sg*FOXP1*, *FOXP1*-targeting sgRNA; sg*Tat*, *Tat*-targeting sgRNA; CRISPR, clustered regularly interspaced short palindromic repeats; Cas9, CRISPR-associated protein 9; SD, standard deviation.

We next chose to further investigate the role of two TFs in HIV latency — GATA3 and FOXP1. Our scRNA-seq data from 2D10 cells had identified the transcript for GATA3 as being positively correlated with HIV vRNA levels, and the scATAC-seq data indicated that the activity of GATA3 was positively correlated with HIV vRNA levels. Furthermore, GATA3-binding motif (MA0037.3) accessibility played a significant role in the performance of our machine learning model of HIV reactivation in 2D10 cells (Figure S21). Additionally, we observed that in primary CD4^+^ T cells, *GATA3* expression was positively correlated with HIV vRNA levels in both donors (Tables S8 and S9). For FOXP1, scATAC-seq data from primary CD4^+^ T cell data demonstrated that the activity of FOXP1 was negatively correlated with HIV vRNA levels for both donors (Tables S8 and S9). When we examined the activity and transcript expression of GATA3 and FOXP1 across the LRA conditions, we observed up-regulation of GATA3 activity/transcript expression and down-regulation of FOXP1 activity/transcript expression within specific LRA-stimulated conditions (**Figure 8**A). Thus, we hypothesize that GATA3 and FOXP1 regulate HIV expression and may play an important role in baseline HIV expression or the response to one or more of the LRAs detected in this study.

We first investigated the role of GATA3 in HIV latency in 2D10 cells by transducing these cells with a *GATA3*-targeting shRNA (sh*GATA3*) lentivirus or a control non-targeting shRNA (shNT) lentivirus. Transduced cells were selected by puromycin, and efficient knockdown of GATA3 was confirmed by Western blot (Figure 8B). Viral gene expression due to transduction in 2D10 cells was then measured by flow cytometry for GFP^+^ signals both at baseline and 24 h after prostratin stimulation. We observed that cells transduced with sh*GATA3* exhibited a significantly reduced response (30.8%) to prostratin compared with cells transduced with shNT (36.9%) (Figure 8C). Although GATA3-independent pathways clearly account for a majority of the response to prostratin, these data demonstrate that GATA3 plays an important accessory role in viral reactivation by this molecule in 2D10 cells.

In contrast with GATA3, we observed that lentiviral transduction of 2D10 cells with a *FOXP1*-targeting shRNA (sh*FOXP1*) showed depletion of FOXP1 by Western blot (Figure 8D) but caused reactivation of vRNA expression (∼ 8% GFP^+^ cells) compared with non-targeting control (∼ 2% GFP^+^ cells) by 5 days post knockdown (Figure 8E and F). To further examine the role of FOXP1 in HIV expression in cell line model of latency, we co-transfected HEK293T cells with a GFP-expressing HIV clone (HIV-GFP) [33] and either a FOXP1-mCherry-expressing plasmid or a mCherry-expressing plasmid (control). Overexpression of FOXP1 isoform D (FOXP1D) in HEK293T cells was confirmed by Western blot (Figure 8G). We noted that the most abundant isoform of endogenous FOXP1 expressed in HEK293T cells was a larger isoform, consistent with the size of FOXP1A. Overexpression of FOXP1D along with HIV in HEK293T cells caused attenuated viral gene expression (51% GFP^+^ cells) compared with the control plasmid (60% GFP^+^ cells) (Figure 8H). Overall, these data indicate that, in cell lines, FOXP1 can play a role in repressing HIV expression.

We also examined the role of GATA3 and FOXP1 in HIV-GFP-infected primary CD4^+^ T cells. To address this, we nucleofected Cas9/single-guide RNA (sgRNA) ribonucleoprotein (RNP) complexes targeting *GATA3* or *FOXP1* into HIV-GFP-infected primary CD4^+^ T cells at 1 week post infection (Figure 8I). Western blot of nucleofected cells confirmed significant depletion of GATA3 and FOXP1 with their respective targeting sgRNAs (Figure 8J, Figure S22A). When we examined HIV expression at 1 week post nucleofection (Figure 8K, Figure S22B), we observed that knockout of *GATA3* or *FOXP1* caused a small reduction in viral gene expression at baseline. We then stimulated the infected cells with 500 nM prostratin for 24 h. This stimulation caused a significant increase in the percentage of GFP^+^ cells in the cell population, indicating latency reversal. While this increase also occurred in GATA3- and FOXP1-depleted cells, the response was attenuated compared with cells nucleofected with a non-targeting Cas9/sgRNA RNP. These data clearly indicate that GATA3 and FOXP1 play accessory roles in the reactivation of HIV expression in response to prostratin treatment in HIV-infected primary CD4^+^ T cells. The divergent behavior of FOXP1 in primary CD4^+^ T cells compared with that in 2D10 cells also indicates that TFs can play complex and sometimes opposing roles in viral gene expression depending on the system/model. Additional investigation will clarify both the repressive and accessory roles of FOXP1.

Overall, these findings highlight the value of the combined multiomics approach to identify and prioritize new candidate regulators of HIV and reveal FOXP1 and GATA3 as previously unappreciated factors that impact the expression of HIV in CD4^+^ T cells. It is likely that additional analyses of HIV-correlated factors derived from this dataset will identify further regulators of HIV transcription. Nevertheless, these data identify GATA3 and FOXP1 as novel and potentially important regulators of HIV expression and reactivation from latency.

## Discussion

Inefficient reactivation of HIV by current LRAs is a major impediment to achieving an HIV cure, and developing methods that can broadly reactivate the replication competent reservoir is an urgent priority [17]. However, the mechanisms responsible for this inefficiency remain largely unknown. Reactivation is regulated in part by stochastic processes relating to fluctuations in the abundance of host cell factors and in the level of the viral *trans*-activator Tat [57,58]. Nevertheless, reactivation is also likely influenced by the overall host cell environment, through the expression or activity of cellular transcriptional regulators that bind and directly regulate the viral LTR, or that influence cell fate decisions that indirectly impact viral gene expression [59]. Previous work from our lab and others has demonstrated that viral silencing in CD4^+^ T cells is not entirely random but is associated with a distinct transcriptomic and epigenomic program [31,44,60,61]. We hypothesize that during the initiation of HIV latency, a set of heritable epigenetic modifications are made to the provirus that maintain viral silencing through cell division, and that the frequency with which these modifications occur is connected to the overall host cell program [27]. Furthermore, this program, in combination with stochastic responses to latency reversing drugs, likely regulates the response of latent proviruses to external latency reversing stimuli [62]. Rather than representing a single state, we propose that latency represents a set of adjacent and interconnected states each characterized by a different set of restrictions to full viral reactivation. The low efficiency of overall reactivation for a given LRA is thus explained by the fact that the pathway targeted by each LRA is limiting only a minor fraction of cells (responders) while for other cells (non-responders) a different set of restrictions continue to block viral expression. As such, detailed investigation of viral reactivation at the single-cell level may reveal additional cellular factors that control reactivation and could be targeted in parallel with current LRAs to greatly improve the overall potency and breadth of LRAs.

Understanding the heterogeneous and dynamic natures of HIV latency will require the application of single-cell methods to model systems of latency in which, ideally, multiple modalities regarding the state of the host cell and the provirus can be simultaneously measured [63]. By combining high dimensional, multimodal observations of latency reversal with analytical methods to connect these observations to viral gene expression, we aim to reveal the complex relationship between host cell factors and viral reactivation. In particular, combining scRNA-seq with scATAC-seq will allow us to connect viral expression to the behavior of cellular chromatin, and, by inference, the activity of individual cellular TFs [64]. Since TFs frequently recruit chromatin remodeling complexes to cellular promoters and enhancers, the re-opening of cellular chromatin in response to stimulation can reveal activity levels for sequence-specific cellular TFs [65]. To date only a handful of publications have used scRNA-seq/scATAC-seq or combined scATAC-seq/surface protein analysis to characterize HIV-infected cells [34,66].

In this study, we reactivated an established cell line and primary CD4^+^ T cell model of latently infected cells with three different LRAs in parallel, using concentrations that produce variegated reactivation of the provirus. As expected, each LRA induced a distinct set of gene expression and TF activity across the population. The overall diversity of transcriptomic and epigenomic phenotypes across the cell population then allowed us to examine the correlation of proviral accessibility and expression with numerous host cell factors. The overall expression of HIV transcripts was significantly correlated with proviral accessibility, consistent with the hypothesis that accessibility of the provirus is a key barrier to reactivation of expression.

By examining the correlation of cellular transcripts and TF activities with HIV vRNA levels, we were able to identify a set of cellular transcripts and TFs that significantly correlated (*P* < 0.05) with vRNA levels in each of these models. Furthermore, we were able to examine the correlation of accessibility of specific chromatin peaks with HIV vRNA levels, and examine these for TF-binding motif enrichment. Altogether, this approach has identified a set of candidate regulators of HIV expression and reactivation that could be investigated further for functional roles in HIV infection. Reassuringly, this approach identified several known HIV regulators of HIV transcription and latency including NF-κB, AP-1, Sp1, CTCF, and MALAT1 [42,67,68]. This observation suggests that this approach is powered to detect regulators of HIV transcription. In addition to these known HIV regulating factors, many genes or TFs with no known connection to HIV transcription were found. These included genes/TFs that were either positively correlated with HIV transcription (candidate activators) or negatively correlated (candidate repressors). Amongst the positively correlated TFs, ETS and GATA families were highly represented. Previous data have linked the activity of some ETS family members to HIV expression, and an ETS-binding site in the HIV 5′ LTR enhancer has been demonstrated [69–73]. Amongst the negatively associated factors, transcriptional repressors of the SNAIL family were identified. No previous reports have connected SNAIL members to HIV gene expression. This family of Zn^2+^ finger TFs has been characterized for their role in regulating epithelial to mesenchymal fate decisions that bind to E-box CANNTG sequences [74]. Notably, the HIV LTR (NL4-3) contains four potential E-box sequences. Additional functional experiments will be needed to confirm whether these novel factors contribute directly or indirectly to HIV gene expression. Through cross-correlation analysis, we have also identified groups of TFs within the HIV-correlated set that are connected to each other, indicating that sets of functionally related TFs can act as part of a coordinated group. As such, carefully defining the roles of individual members of these groups will be important.

Notably, the overall correlations of individual TFs and transcripts, although statistically significant, were weak. We speculate that these low correlations result from the additional influence of background noise or stochastic processes that also contribute to reactivation. Alternatively, this observation could result from latency reversal being determined by the combined activity of several different host cell factors. To address this possibility, we used a machine learning approach to develop a multivariate model to predict whether individual cells expressed vRNAs across the dataset. We successfully developed a boosting-based model with ∼ 75%–79% accuracy to classify cells highly expressing vRNAs (top 10%), confirming that a machine learning approach improves the overall understanding of viral reactivation.

To validate this overall approach, we chose to focus on the functional roles of two cellular factors, GATA3 and FOXP1. Although a previous report has shown that GATA3 can bind and activate an LTR-driven reporter plasmid in an epithelial cell line, the role of GATA3 in HIV transcription and latency is unknown [75]. GATA3 was identified by both correlations of the GATA3 transcript and GATA3-binding motif accessibility with vRNA levels. Across the experimental conditions, GATA3 transcript expression and activity were most clearly induced by prostratin, suggesting a possible role for GATA3 in regulating reactivation of HIV latency by prostratin. Selective knockdown of *GATA3* in 2D10 cells and in primary CD4^+^ T cells diminished the ability of prostratin to reactivate HIV. Although, clearly, prostratin is not strictly dependent on GATA3 for its latency reversing activity, we propose that GATA3 induction represents one of several pathways activated by prostratin in CD4^+^ T cells that ultimately combine to promote HIV reactivation. As such, a GATA3-targeting drug could potentially be combined with prostratin or other LRAs to enhance reservoir reactivation during latency reversal therapy. To date, toxicity concerns have limited enthusiasm for PKC agonists such as prostratin in therapy [76], but a combined stimulation of a PKC agonist with a GATA3 agonist could potentially permit a lower and safer dose of prostratin to be used. Conversely, a GATA3 inhibitor such as pyrrothiogatain could be used to block viral reactivation and promote deeper latency [77]. An issue of concern with this approach is that GATA3 plays important physiological roles in promoting the development of important T cell subsets [78]. It also remains to be determined whether the role of GATA3 in this response is direct or indirect.

Our data also suggest that FOXP1 represents a novel regulator of HIV expression. FOXP1 activity was negatively correlated with vRNA levels, leading us to hypothesize that FOXP1 could repress HIV transcription. Consistent with this hypothesis, knockdown of *FOXP1* in 2D10 cells reactivated viral gene expression, and overexpression of FOXP1D inhibited HIV expression in HEK293T cells. Curiously, in primary CD4^+^ T cells, knockout of *FOXP1* diminished HIV expression after prostratin stimulation, suggesting that this factor may play opposing roles in different cell models of HIV latency. Additional studies will be required to resolve this issue. Notably, a previous study has identified another forkhead TF (FOXO1) as a repressor of HIV, suggesting that multiple FOX TFs may participate in regulating HIV expression [79].

This study should also be considered in light of several inherent limitations and caveats. Firstly, 2D10 cells are an immortalized cell line which have several differences with primary resting CD4^+^ T cells, the natural host cell for the HIV reservoir. Furthermore, the proviral clones used for both the 2D10 cell and the primary CD4^+^ T cell latency models are not complete HIV strains and have inactivating mutations in several viral proteins that could conceivably also contribute to the behavior of the virus in the context of latency reversal. It is also important to note that our approach rested on examining correlations between cellular factors and viral transcription, with the assumption that some of these correlations represent functional contributors to viral expression. However, it is also possible that while correlated, some factors may be induced by the LRAs tested, but not be causally linked to viral gene reactivation. Other correlated factors may also represent non-contributing “bystanders”. Nevertheless, the confirmation of the functional roles of GATA3 and FOXP1 demonstrates the power of this approach to identify novel host–viral connections that could be exploited to enhance latency reversal approaches.

## Materials and methods

### Reactivation of latently infected 2D10 cell line

A latently infected Jurkat derived T cell line (2D10 cells) was cultured in Roswell Park Memorial Institute (RPMI) 1640 medium supplemented with 10% fetal bovine serum (FBS), penicillin (100 IU/ml), streptomycin (100 μg/ml), and 25 mM 4-(2-hydroxyethyl)-1-piperazineethanesulfonic acid (HEPES) buffer at 37°C in 5% CO_2_. The 2D10 cell line carries an HIV provirus that expresses a short-lived green fluorescent protein (d2EGFP) in place of Nef [80]. The latently infected cells were reactivated with three LRAs with different mechanisms of action, prostratin (PKCa), iBET151 (BRDi), or vorinostat (HDACi). Concentrations used for reactivation of the virus were 75 nM for prostratin and iBET151 and 500 nM for vorinostat. After 24 h treatment, reactivated 2D10 cells were quantified for GFP expression by BD LSRFortessa flow cytometry (Becton Dickson, Franklin Lakes, NJ) and analyzed using FlowJo (version X10.0.7r2).

### Primary CD4^+^ T cell model of HIV latency

CD4^+^ T cells were isolated from uninfected donor peripheral blood mononuclear cells (PBMCs) using the EasySep Human CD4^+^ T Cell Isolation Kit (Catalog No. 17952, StemCell Technologies, Vancouver, Canada) using negative selection following the manufacturer’s protocol. Purity of CD4^+^ cells were determined by flow cytometry, which showed a high purity (97.5%) CD4^+^ T cell population. Cells were plated at 1 × 10^6^ cells/ml in RPMI supplemented with 10% FBS and penicillin/streptomycin, and incubated overnight at 37°C. The cells were activated using Dynabeads Human T-Activator CD3/CD28 beads (Catalog No. 111.31D, Thermo Fisher Scientific, Waltham, MA) for 72 h at a ratio of 1:1 (beads:cells). Dynabeads were then removed, and activated cells were spin-infected with NL4-3-△6-dreGFP-IRES-thy1.2 virus [81,82] at a speed of 600 *g* for 2 h. Successful infection was confirmed by measuring GFP expression by flow cytometry at 2 days post infection (dpi) followed by enrichment of infected cells by flow sorting using a BD FACSAria II (Becton Dickinson, Franklin Lakes, NJ). Enriched cells expressing GFP were kept in culture for 3 weeks, during which time viral gene expression diminished, leaving most infected cells GFP^−^. The GFP^−^ population was then re-sorted to enrich the latently infected population. Latently infected cells were then treated with the LRAs, vorinostat, prostratin, or iBET151, at concentration of 500 nM along with vehicle control (DMSO, 0.05%). After 24 h treatment, cells were harvested, washed, and quantified for reactivation (GFP) by flow cytometry. The remainder of the reactivated cells were used for integrated single-cell multiomic scRNA-seq/scATAC-seq library preparation.

### Nuclei isolation for scRNA-seq/scATAC-seq

Nuclei were isolated following the 10X Genomics protocol (CG000365 • Rev C) with slight modifications. Cells were harvested, washed once with ice-cold phosphate buffered saline (PBS) supplemented with 0.5% bovine serum albumin (BSA) and 0.2 U/μl RiboLock RNase Inhibitor (Catalog No. EO0382, Thermo Fisher Scientific), counted, and viability detected by Trypan Blue (Catalog No.15250061, Thermo Fisher Scientific). Dead cells were first removed using Dead Cell Removal Kit (Catalog No. 130-090-101, Miltenyi Biotec, Bergisch Gladbach, Germany). For each sample, 1 × 10^6^ cells were pelleted by centrifugation at 500 *g*, 4°C, 5 min. The cells were resuspended in 100 μl of ice-cold lysis buffer [10 mM Tris-HCl pH 7.4, 10 mM NaCl, 3 mM MgCl_2_, 0.01% Tween 20 (Catalog No. 655205-250ML, Millipore Sigma, Burlington, MA), 0.01% nonidet P40 substitute (Catalog No. I8896, Millipore Sigma), 0.01% digitonin (Catalog No. G9441, Promega, Madison, WI), 1 U/μl RiboLock RNase Inhibitor, 1% BSA, and 1 mM dithiothreitol (DTT)] and incubated on ice for 3 min. The nuclei were then pelleted, washed with 1 ml ice-cold wash buffer (10 mM Tris-HCl pH 7.4, 10 mM NaCl, 3 mM MgCl_2_, 0.01% Tween 20, 1% BSA, 1 mM DTT, and 1 U/μl RiboLock RNase Inhibitor), and re-suspended in diluted nuclei buffer (1× nuclei buffer, 1 mM DTT, and 1 U/μl RiboLock RNase Inhibitor).

### Single-cell multiome (scRNA-seq and scATAC-seq) library construction and sequencing

Construction of single-cell multiome libraries was performed using a 10X Genomics Chromium Controller and a Single Cell Multiome ATAC + Gene Expression kit (Catalog No. PN-1000285, 10X Genomics, Pleasanton, CA) following manufacturer’s protocol (CG000338 • Rev D). Nuclei were first isolated following the 10X Genomics protocol (CG000365 • Rev B) as described above. Then, the intact nuclei suspensions were subjected to Tn5 transposition in bulk, followed by barcoding using Gel Beads-in-emulsion (GEM) beads. Silane magnetic beads were used to purify the barcoded products from the post GEM-Reverse Transcription (RT) reaction mixture. Incubation of the GEMs produces barcoded DNA from the transposed DNA (for scATAC-seq) and barcoded, full-length complementary DNA (cDNA) from poly-adenylated messenger RNA (mRNA) (for scRNA-seq). Barcoded transposed DNA and barcoded full-length cDNA were then pre-amplified by PCR to facilitate library construction. The pre-amplified product was used as input for both ATAC-seq and RNA-seq library constructions. P5 and P7 indexes were added to the pre-amplified transposed DNA for ATAC-seq libraries. cDNA amplification, enzymatic fragmentation followed by end repair, A-tailing, adaptor ligation, and PCR were performed to incorporate P5, P7, i7, and i5 sample indices, and TruSeq Read 2 (read 2 primer sequence) for RNA-seq libraries. The libraries were quantified using an Agilent TapeStation 4200 (Agilent Technologies, Santa Clara, CA) and the Qubit dsDNA High Sensitivity Assay Kit (Catalog No. Q33230, Invitrogen, Waltham, MA). Pooled samples from ATAC-seq and RNA-seq libraries were sequenced using paired-end, single-index (ATAC-seq) and dual index (RNA-seq) sequencing on a NextSeq 2000 instrument (Illumina, San Diego, CA). For RNA-seq libraries, the read format was: Read 1 — 28 cycles; Read 2 — 90 cycles; i7 — 10 cycles; i5 — 10 cycles. For ATAC-seq libraries, the read format was: Read 1 — 50 cycles; Read 2 — 49 cycles; i7 — 8 cycles; i5 — 16 cycles. Paired-end reads of pooled libraries were demultiplexed prior to downstream analysis.

### scRNA-seq and scATAC-seq data processing

For the 10X Genomics multiome data, we used Cell Ranger ARC (version 2.0.0) to perform sample demultiplexing, barcode processing, peak calling, and counting of RNA-seq and ATAC-seq reads in single cells. HIV–human reference genome was generated based on Sanger sequencing of the NL4-3-△6-dreGFP-IRES-thy1.2 reporter plasmid (described above). The 3′ LTR was masked to prevent issues with multi-mapping. A fasta file representing the viral genome was concatenated to a GRCh38 human reference genome, and a Cell Ranger compatible reference genome was generated using the cellranger-arc mkref function. Alignment was performed against this reference genome with the cellranger-arc count function. To validate our HIV-specific mapping, we analyzed scRNA-seq and scATAC-seq data from uninfected PBMCs using our HIV–human reference genome. The uninfected dataset used was retrieved from 10X Genomics (https://www.10xgenomics.com/resources/datasets/10-k-human-pbm-cs-multiome-v-1-0-chromium-controller-1-standard-2-0-0) covering 57,425 mean raw read pairs per cell for ATAC-seq and 54,464 mean raw reads per cell for RNA-seq, respectively. Using this dataset, we did not observe any HIV mapping reads in uninfected PBMCs, demonstrating that the viral data do indeed reflect genuine information about HIV expression and accessibility.

Cells with high percentages of mitochondrial reads, extreme RNA-seq read counts, and/or extreme ATAC-seq fragment counts were removed (see quality control metrics before and after cell filtering in Figure S3). Doublet cells, identified by scDblFinder across multiplexed samples corresponding to the treatment groups, were excluded from subsequent analysis [83]. For read count normalization, we used sctransform by Seurat [64] for scRNA-seq and term frequency-inverse document frequency (TF-IDF) by Signac [53] for scATAC-seq. This was followed by principal component analysis (PCA) and latent semantic indexing (LSI) for dimension reduction, respectively. We kept the top 50 PCs from scRNA-seq and the top 2–50 LSIs from scATAC-seq. Jointly, these are used as input to construct a weighted nearest neighbor (WNN) graph, which is further used for joint UMAP [32] visualization and clustering. We used the Leiden clustering algorithm for community detection with the resolution parameter visualized and optimized by the clustree algorithm [84], which builds a tree to visualize and examine how clusters are related to each other at varying resolutions, allowing researchers to assess which clusters are distinct and which are unstable with the use of additional metrics such as the SC3 stability index [85]. For modality-specific processing, we built the unweighted K-nearest neighbor (KNN) and the weighted shared nearest neighbor (SNN) graphs, followed by UMAP visualization and clustering identification. For scATAC-seq data, we also adopted weighted PCA by Destin [86] to weigh chromatin accessibilities by existing genomic annotations and publicly available regulomic data.

We obtained the position frequency matrices and annotated 633 pairs of TFs and motifs from the JASPAR 2020 database [87]; we further applied chromVAR [40] to derive, for each TF, its motif score, which measures the deviation in chromatin accessibility across the set of peaks containing the corresponding motif, compared with a set of background peaks. We also constructed a pseudo-gene activity matrix [88], summing ATAC-seq read counts in gene bodies and promoter regions (2 kb upstream of genes’ TSSs), followed by sctransform normalization.

### Differential gene expression and motif accessibility

For each treated group (iBET151, prostratin, and vorinostat), we performed a nonparametric Wilcoxon rank sum test to identify DEGs and differentially accessible motifs compared to the DMSO control. We used the normalized gene expression levels by sctransform and the motif deviation scores by chromVAR as input, and adopted FDR for multiple testing correction. Cauchy combination testing [89] was performed to integrate the three sets of condition-specific *P* values that are correlated. For significant DEGs, we carried out a GO enrichment analysis using the Database for Annotation, Visualization and Integrated Discovery (DAVID) [90].

### Gene, TF, and peak linkage analyses

For HIV expression, we calculated, for each cell, the proportion of HIV vRNA reads out of the total number of reads. For HIV chromatin accessibility, we directly used the total number of HIV ATAC-seq reads, due to the sparsity of the scATAC-seq data. We used the square root (sqrt) of the proportion of HIV vRNA reads as the outcome variable in various supervised frameworks, aiming to recover the molecular biomarkers that are related to HIV expression upon LRA treatment and thus latency reversal. The dependent variables in such linkage analyses include gene expression, TF motif scores, and peak accessibilities. For gene and TF linkage analyses, we adopted *Q*-value based FDR to control for false positives and to adjust *P* values for multiple testing correction [91]. Due to the large number of testing in the peak linkage analysis and the low power due to the ATAC sparsity, we used a significance threshold of 0.01. For the significantly linked peaks, we further searched for motifs that were overrepresented in them compared with a background peak set using the hypergeometric test of enrichment.

### TF network analysis

For TF-specific testing from the TF expression linkage analysis, motif enrichment analysis, and differential accessibility analysis, we compared the calling results and generated a master output to identify key TF regulators associated with latency reversal. We performed consensus PCA to jointly reduce dimensions of TF-coding gene expression levels and deviation scores of TF-binding motifs. For each pair of TFs, we calculated the correlation coefficients between their consensus PCs and constructed an adjacency matrix for the TF network by creating an edge between two TFs if the correlation between them is greater than 0.5.

### Machine learning model of HIV reactivation

Due to the large number of features in the dataset, feature selection is necessary to create an interpretable and usable set of features for a machine learning model. In order to select a high-performance subset of features for prediction, we first calculated Spearman correlation coefficients between each feature within the multiomic datasets against the HIV gene expression before binarization of the cell data to HIV+ or HIV− based on the presence or absence of any detectable vRNA for 2D10 cells or ranking above or below the 90th percentile for HIV vRNA levels for primary CD4^+^ T cells. From each feature set (RNA expression, motifs, and peaks), features with top *k* ranking Spearman correlation coefficients were then selected for modeling. Here, *k* is a hyperparameter to be determined by parameter tuning. For 2D10 cells, we performed parameter tuning, choosing 30 features from RNA expression, 20 from motifs, and 10 from peaks that were then used to train a gradient boosting tree classifier, which constructed a strong final classifier by combining several weaker classifiers. In gradient boosting, weak learners were optimized in an iterative fashion with loss functions that focus on the residuals (errors) of their predecessors. Here we used LightGBM, an efficient implementation of this algorithm [92]. The parameters of this model were then tuned via 10-fold cross-validation and grid search. The feature importance in LightGBM was calculated as the total number of splits of each feature in all trees and was used in our experiment to rank each selected input feature.

For primary CD4^+^ T cells, the initial modeling feature set consisted of the RNA expression and TF activity features observed across both donors (*n* = 8497). For both the XGBoost models and GOSDT models, 80% of the data was randomly selected as a training set, while a test set of 18,508 was held out to evaluate the performance of the final models. Using the training data, absolute Spearman correlation coefficients were calculated between individual features and the vRNA label. The highest-ranked 1000 features by absolute Spearman correlation coefficients were considered for further modeling analysis. These 1000 features excluded mitochondrial features. Next, vRNA was binarized at a split value of 52, where cells with a vRNA value greater than 52 represented the top 10% of the overall data, while those with a vRNA value less than or equal to 52 comprised the bottom 90%.

XGBoost models [56] were trained directly using a binary loss objective through the XGBoost python package (version 1.6.2). Models that trained only using data from one donor only used the subset of the training data coming from that donor. Training involved hyperparameter tuning via a randomized grid search procedure with 100 iterations and 5-fold cross-validation of the training set using an objective of average precision. The hyperparameters tuned include the number of tree estimators, tree depth, subsampling ratio, column sample ratio, label weights, learning rate (eta), and the minimum number of observations. A complete list of hyperparameter thresholds chosen can be found at https://github.com/CalebKornfein/latency. Feature importance was ranked using permutation testing on the test set. Each feature was randomly shuffled five times, and the features were ranked by average drop in test area under the precision–recall curve.

GOSDT models [54] are a class of interpretable, optimal, and sparse decision trees. As with XGBoost, the top 1000 non-mitochondrial features ranked by absolute Spearman correlation coefficients were considered. The feature set of continuous features was reduced into a small set of binarized features using the threshold guessing column elimination procedure proposed by McTavish and his colleagues [55]. This procedure iteratively drops columns of least importance as determined by a black-box boosting model, in our case scikit-learn’s implementation of a gradient boosting classifier. A stopping condition for backward selection was met when both (1) further dropping resulted in deviance performance loss of the gradient boosting classifier and (2) the number of remaining thresholds to binarize features was less than or equal to 30. We varied hyperparameters of the gradient boosting classifier in the threshold guessing procedure including the number of estimators and the weight of the sample labels by randomly oversampling the minority class. Finally, GOSDT models were trained on the remaining binary splits using the python package gosdt (version 0.1.7). GOSDT trees were created for a variety of tree depths, regularization levels, and relative sample weights. Between varying the thresholds and gosdt hyperparameters, 2258 models were produced.

### CRISPR/Cas9-mediated knockout of *GATA3* and *FOXP1* in latently infected primary CD4^**+**^ T cells

Pre-designed CRISPR RNAs (crRNAs) against *GATA3* (5′-GGAGCTGTACTCGGGCACGT-3′ and 5′-TCGACGAGGAGGCTCCACCC-3′), *FOXP1* (5′-ACGGTTCAGCCATCCAGAAT-3′, 5′-GTCATCATAGCCACTGACAC-3′, and 5′-GTGGATTAAAATCTCCCAAG-3′), *Tat* (5′-CCTTAGGCATCTCCTATGGC-3′), and scrambled guides (non-targeting sgRNAs) were obtained from Integrated DNA Technologies (IDT). Annealing of crRNA and trans-activating CRISPR RNA (tracrRNA) to generate sgRNAs and preparing Cas9/sgRNA RNP complexes were performed as described previously [82,93]. Two or three sgRNAs targeting different regions of the target were multiplexed for more efficient target knockout. For each electroporation, latently infected primary CD4^+^ T cells were washed twice with PBS at a speed of 90 *g* for 10 min, and then resuspended in nucleofection buffer P3 (Catalog No. V4SP-3096, Lonza Bioscience, Basal, Switzerland) at a concentration of 1.5 × 10^6^ cells/ml. The resuspended cells were combined with Cas9/sgRNA RNP complexes, immediately transferred into the cuvette of the P3 Primary Cell 96-well Nucleofector Kit (Catalog No. V4SP-3096, Lonza Bioscience), and electroporated using code CM-137 on the Lonza 4D-Nucleofector Cell Transfection System Core Unit and X-Unit (Newlife Scientific, Ohio, IL). Electroporated cells were resuspended with 200 μl of prewarmed RPMI media supplemented with interleukin (IL)-2 (20 U/ml) and IL-7 (5 ng/ml), and expanded in a 37°C incubator for subsequent experiments. The cells were maintained at 1 × 10^6^ cells/ml with fresh media supplemented with IL-2 and IL-7 every 2–3 days. A week after nucleofection, knockout cells were treated with DMSO or 500 nM prostratin for 24 h, and the expression of GFP was quantified by flow cytometry.

### shRNA knockdown and overexpression

For knockdown experiments, lentiviruses encoding shRNAs against *FOXP1*, *GATA3*, or scrambled control were generated by co-transfecting viral expression plasmids with Gag-Pol and VSV-G packaging plasmids into HEK293T cells. Virus containing supernatant was subjected to low-speed centrifugation (300 *g*, 5 min) and filtration using a 0.45-μm filter to removed cellular contaminants. The remaining supernatant was added to 2D10 cells in complete RPMI media for 24 h to facilitate infection. For *GATA3* knockdown, the targeting lentivirus encoded a puromycin resistance gene, and transduced cells were selected by inclusion of 1μg/ml puromycin in the cell media. For *FOXP1* knockdown, the targeting lentivirus also encoded an mCherry expression cassette, and transduced cells were distinguished from non-transduced cells by flow cytometry and gating for mCherry^+^ cells. Five days post transduction, cells were quantified for viral gene expression (GFP) by flow cytometry.

For overexpression of *FOXP1*, plasmids encoding a FOXP1-mCherry fusion separated by a 2A peptide or mCherry only were co-transfected with NL4-3-△6-dreGFP-IRES-thy1.2 into HEK293T cells. Three days post transfection, GFP expression in the mCherry^+^ population was quantified. Cells from knockdown and overexpression experiments were also harvested for Western blot analysis.

### Western blot

To extract cellular protein for Western blot, cells were lysed using radioimmunoprecipitation assay (RIPA) buffer. Lysates were resolved on a Novex WedgeWell 4%–20% Tris–Glycine gel (Catalog No. XP04202BOX, Invitrogen) and transferred to nitrocellulose membranes (Catalog No. IB23002, Invitrogen). Blocking was performed for 1 h at room temperature using 5% milk in Tris-buffered saline (TBS). Immunoblotting was performed using the primary antibodies: anti-GATA3 (1:1000; Catalog No. ab106625, Abcam, Cambridge, UK), anti-FOXP1 (1:1000; Catalog No. ab134055, Abcam), and anti-β-actin (1:10000; Catalog No. ab49900, Abcam). Membranes were washed with TBS-tween (TBST) 3 times for 10 min each. The donkey anti-rabbit IgG-HRP (1:10000; Catalog No. A16035, Invitrogen) was used as the secondary antibody. Membranes were developed with ECL Prime Western Blotting Detection Reagents (Catalog No. RPN2232, Cytiva, Marlborough, MA) and visualized on a Bio-Rad ChemiDoc MP Imaging System (Bio-Rad, Hercules, CA).

## Supplementary Material

qzae003_Supplementary_Data

## Data Availability

The data reported in this study have been deposited in Gene Expression Omnibus (GEO: GSE242997 for primary CD4^+^ T cells, GSE210146 for 2D10 cell line), and are publicly accessible at https://www.ncbi.nlm.nih.gov/geo.

## Code availability

Scripts and reference genomes used for analyses carried out in this study have been deposited in the GitHub repository at https://github.com/yuchaojiang/HIV-latency.

## CRediT author statement

**Manickam Ashokkumar:** Methodology, Validation, Formal analysis, Investigation, Data curation, Writing – original draft, Visualization. **Wenwen Mei:** Methodology, Software, Formal analysis, Data curation, Writing – review & editing, Visualization. **Jackson J. Peterson:** Methodology, Software, Formal analysis, Investigation, Data curation, Writing – review & editing. **Yuriko Harigaya:** Methodology, Software, Formal analysis, Visualization. **David M. Murdoch:** Methodology, Resources. **David M. Margolis:** Methodology, Resources, Funding acquisition. **Caleb Kornfein:** Methodology, Software, Formal analysis, Visualization. **Alex Oesterling:** Methodology, Software, Formal analysis, Visualization. **Zhicheng Guo:** Methodology, Software, Formal analysis, Visualization. **Cynthia D. Rudin:** Methodology, Software, Formal analysis, Resources, Visualization, Supervision, Funding acquisition. **Yuchao Jiang:** Methodology, Software, Formal analysis, Resources, Data curation, Writing – original draft, Visualization, Supervision, Project administration, Funding acquisition. **Edward P. Browne:** Conceptualization, Methodology, Validation, Formal analysis, Resources, Writing – original draft, Supervision, Project administration, Funding acquisition. All authors have read and approved the final manuscript.
