# Supplementary material for: Integrated Single-cell Multiomic Analysis of HIV Latency Reversal Reveals Novel Regulators of Viral Reactivation
Source: Genomics Proteomics Bioinformatics. 2024 Jun 20;22(1):qzae003. doi: 10.1093/gpbjnl/qzae003 (PMC11189801; doi:10.1093/gpbjnl/qzae003)
Supplement: qzae003_Supplementary_Data [file qzae003_supplementary_data.zip › Figure S10.pdf]

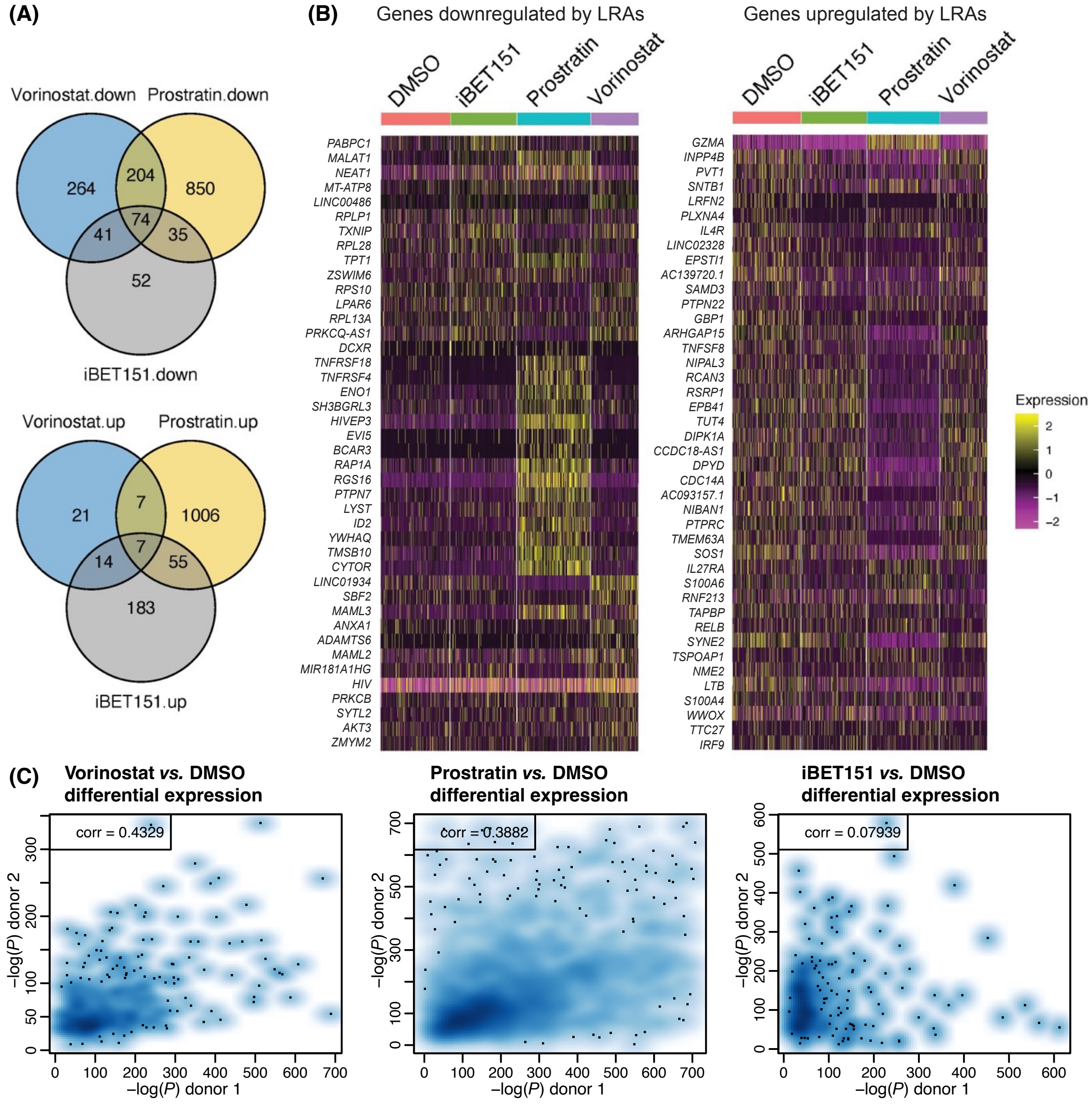
(A)
Vorinostat.down
Prostratin.down
264
204
850
74
41
35
52
iBET151.down
Vorinostat.up
Prostratin.up
21
7
1006
7
14
55
183
iBET151.up
(B)
Genes downregulated by LRAs
Genes upregulated by LRAs
DMSO
iBET151
Prostratin
Vorinostat
PABPC1
MALAT1
NEAT1
MT-ATP8
LINC00486
RPLP1
TXNIP
RPL28
TPT1
ZSWIM6
RPS10
LPAR6
RPL13A
PRKCQ-AS1
DCXR
TNFRSF18
TNFRSF4
ENO1
SH3BGRL3
HIVEP3
EVI5
BCAR3
RAP1A
RGS16
PTPN7
LYST
ID2
YWHAQ
TMSB10
CYTOR
LINC01934
SBF2
MAML3
ANXA1
ADAMTS6
MAML2
MIR181A1HG
HIV
PRKCB
SYTL2
AKT3
ZMYM2
GZMA
INPP4B
PVT1
SNTB1
LRFN2
PLXNA4
IL4R
LINC02328
EPSTI1
AC139720.1
SAMD3
PTPN22
GBP1
ARHGAP15
TNFSF8
NIPAL3
RCAN3
RSRP1
EPB41
TUT4
DIPK1A
CCDC18-AS1
DPYD
CDC14A
AC093157.1
NIBAN1
PTPRC
TMEM63A
SOS1
IL27RA
S100A6
RNF213
TAPBP
RELB
SYNE2
TSPOAP1
NME2
LTB
S100A4
WWOX
TTC27
IRF9
Expression
2
1
0
−1
−2
(C)
Vorinostat vs. DMSO differential expression
corr = 0.4329
−log(P) donor 2
−log(P) donor 1
Prostratin vs. DMSO differential expression
corr = 0.3882
iBET151 vs. DMSO differential expression
corr = 0.07939
