# Supplementary material for: Integrated Single-cell Multiomic Analysis of HIV Latency Reversal Reveals Novel Regulators of Viral Reactivation
Source: Genomics Proteomics Bioinformatics. 2024 Jun 20;22(1):qzae003. doi: 10.1093/gpbjnl/qzae003 (PMC11189801; doi:10.1093/gpbjnl/qzae003)

(A) Aggregated *vs.* condition-specific gene-linkage analyses

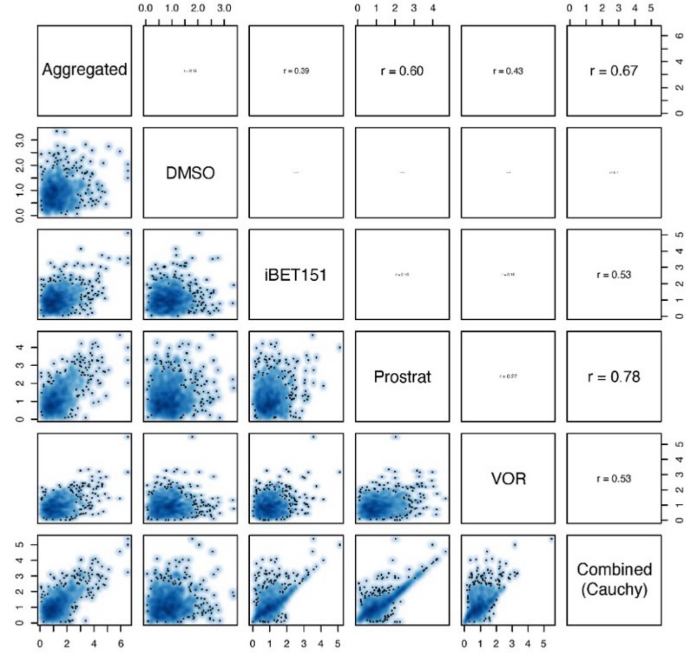

(B) Aggregated *vs.* condition-specific TF-linkage analyses

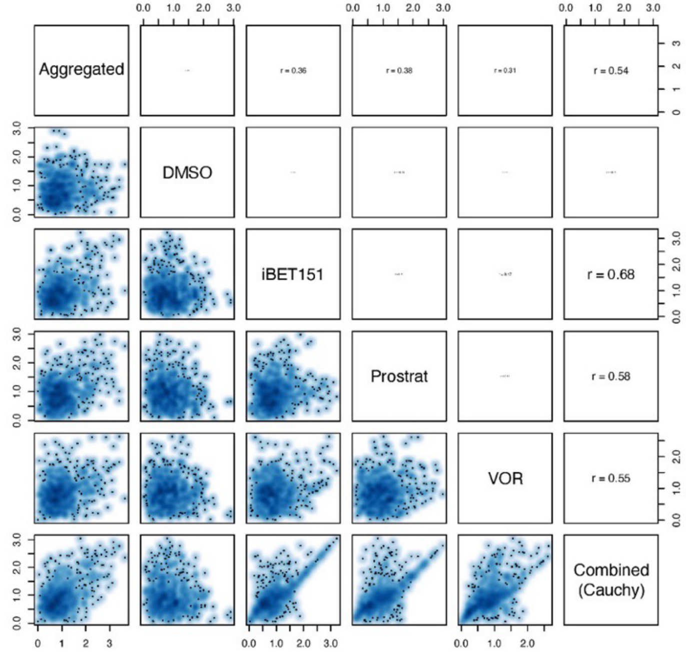

Supplement: qzae003_Supplementary_Data [file qzae003_supplementary_data.zip › Figure S16.pdf]
