# Supplementary material for: Integrated Single-cell Multiomic Analysis of HIV Latency Reversal Reveals Novel Regulators of Viral Reactivation
Source: Genomics Proteomics Bioinformatics. 2024 Jun 20;22(1):qzae003. doi: 10.1093/gpbjnl/qzae003 (PMC11189801; doi:10.1093/gpbjnl/qzae003)
Supplement: qzae003_Supplementary_Data [file qzae003_supplementary_data.zip › Figure S17.pdf]

(A)

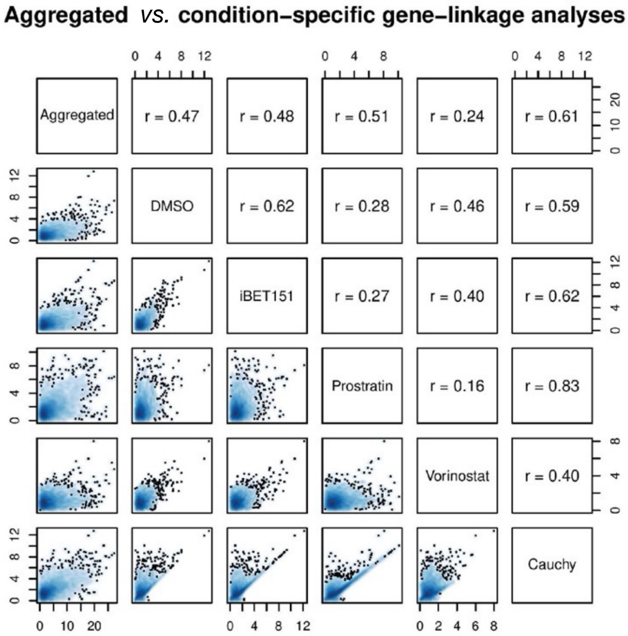
Aggregated vs. condition-specific gene-linkage analyses
Aggregated
DMSO
iBET151
Prostratin
Vorinostat
Cauchy
r = 0.47
r = 0.48
r = 0.51
r = 0.24
r = 0.61
r = 0.62
r = 0.28
r = 0.46
r = 0.59
r = 0.27
r = 0.40
r = 0.62
r = 0.16
r = 0.83
r = 0.40

(B)

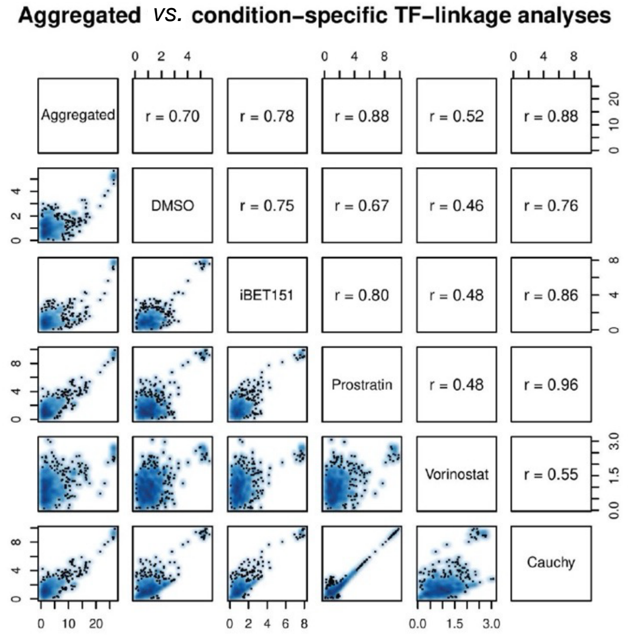
Aggregated vs. condition-specific TF-linkage analyses
Aggregated
DMSO
iBET151
Prostratin
Vorinostat
Cauchy
r = 0.70
r = 0.78
r = 0.88
r = 0.52
r = 0.88
r = 0.75
r = 0.67
r = 0.46
r = 0.76
r = 0.80
r = 0.48
r = 0.86
r = 0.48
r = 0.96
r = 0.55
