# Supplementary material for: Integrated Single-cell Multiomic Analysis of HIV Latency Reversal Reveals Novel Regulators of Viral Reactivation
Source: Genomics Proteomics Bioinformatics. 2024 Jun 20;22(1):qzae003. doi: 10.1093/gpbjnl/qzae003 (PMC11189801; doi:10.1093/gpbjnl/qzae003)
Supplement: qzae003_Supplementary_Data [file qzae003_supplementary_data.zip › Figure S18.pdf]

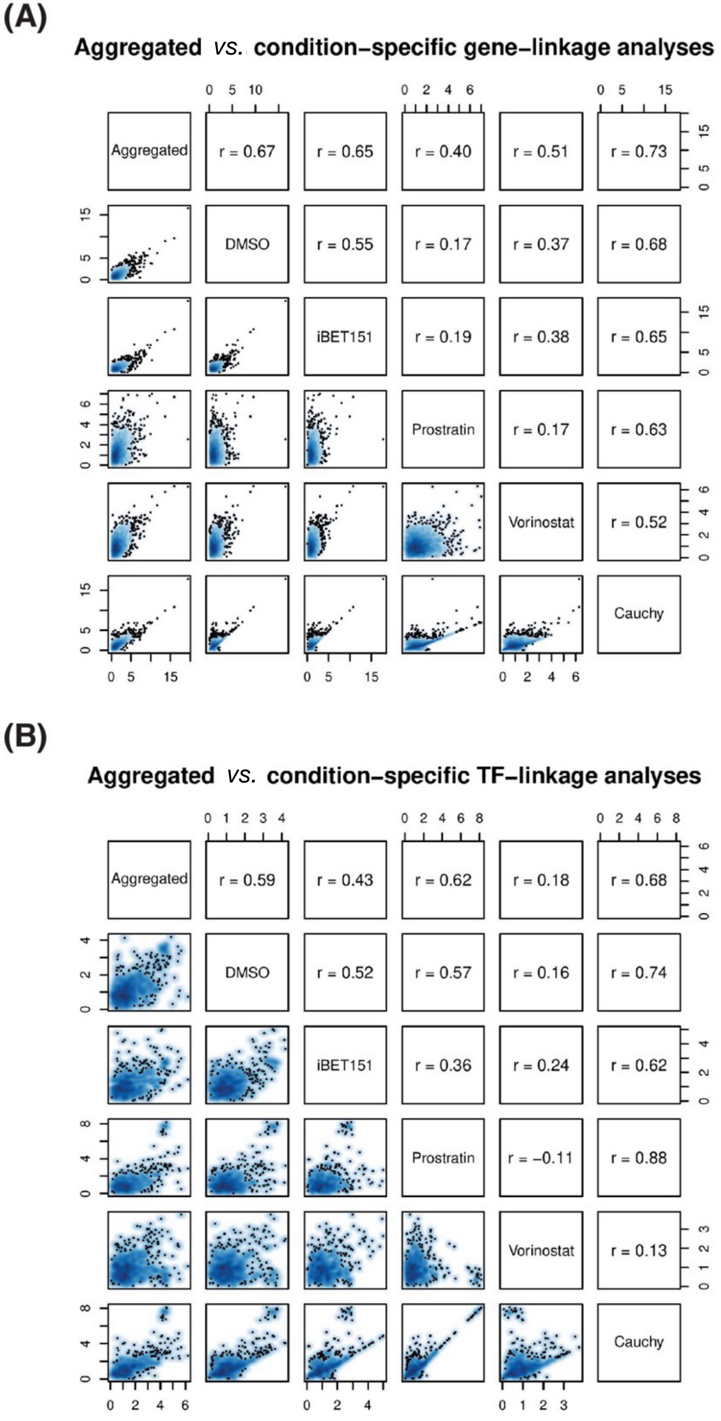

(A)
Aggregated vs. condition-specific gene-linkage analyses
Aggregated
DMSO
iBET151
Prostratin
Vorinostat
Cauchy
r = 0.67
r = 0.65
r = 0.40
r = 0.51
r = 0.73
r = 0.55
r = 0.17
r = 0.37
r = 0.68
r = 0.19
r = 0.38
r = 0.65
r = 0.17
r = 0.63
r = 0.52
(B)
Aggregated vs. condition-specific TF-linkage analyses
Aggregated
DMSO
iBET151
Prostratin
Vorinostat
Cauchy
r = 0.59
r = 0.43
r = 0.62
r = 0.18
r = 0.68
r = 0.52
r = 0.57
r = 0.16
r = 0.74
r = 0.36
r = 0.24
r = 0.62
r = −0.11
r = 0.88
r = 0.13
