# Supplementary material for: Integrated Single-cell Multiomic Analysis of HIV Latency Reversal Reveals Novel Regulators of Viral Reactivation
Source: Genomics Proteomics Bioinformatics. 2024 Jun 20;22(1):qzae003. doi: 10.1093/gpbjnl/qzae003 (PMC11189801; doi:10.1093/gpbjnl/qzae003)
Supplement: qzae003_Supplementary_Data [file qzae003_supplementary_data.zip › Supplementary captions.docx]

**Supplementary material**

**Figure S1 Single-cell multiomic analysis of HIV latency reversal in 2D10 cells**

**A.** A schematic of the experimental design of HIV latency reversal followed by multiomic scRNA-seq and scATAC-seq. **B.** Percentage of cells expressing the GFP reporter in the control (DMSO), as well as in those treated with LRAs (vorinostat, prostratin, and iBET151). **C.** UMAP dimension reduction of scRNA-seq, scATAC-seq, and WNN with cells labeled by conditions. **D.** UMAP dimension reduction of scRNA-seq, scATAC-seq, and WNN with cells labeled by cluster. HDACi, histone deacetylase inhibitor; BRDi, bromodomain inhibitor; PKCa, protein kinase C activator.

**Figure S2 Single cell multiomic analysis of HIV latency reversal for primary cell donor 2**

**A.** A schematic of the experimental design of primary CD4 T cell HIV latency model followed by stimulation with LRAs. **B.** Flow cytometry dot plot showing the viral GFP expression in the control cells (DMSO), as well as in cells treated with LRAs (vorinostat, prostratin, and iBET151) for donor 2. **C.** Bar plot showing percentage of GFP+ cells with and without stimulation by iBET151, vorinostat, and prostratin. **D.** UMAP dimension reduction of scRNA-seq, scATAC-seq, and WNN with cells labeled by conditions. **E.** UMAP dimension reduction of scRNA-seq, scATAC-seq, and WNN with cells labeled by graph-based clusters.

**Figure S3 Quality control of the scRNA-seq and scATAC-seq data**

**A.** Violin plots showing distribution of scATAC-seq read counts per cell (left), scRNA-seq read counts per cell (middle), and scRNA-seq read counts mapping to the mitochondrial genome (right) for 2D10 cell line data. The left and right panel correspond to the data before and after filtering. QC metrics adopted: number of ATAC reads between 1000 and 20,000; number of RNA reads between 1000 and 25,000; percentage of mitochondrial reads below 20%. **B.** The same as A but for primary CD4 T cells from donor 1. QC metrics adopted: number of ATAC reads between 1000 and 25,000; number of RNA reads between 1000 and 10,000; percentage of mitochondrial reads below 20%. **C.** The same as A but for primary CD4 cells from donor 2. QC metrics adopted: number of ATAC reads between 1000 and 30,000; number of RNA reads between 1000 and 10,000; percentage of mitochondrial reads below 20%. QC, quality control.

**Figure S4 HIV viral RNA expression and chromatin accessibility in 2D10 cells**

**A.** Transformed HIV RNA reads under different treatment conditions. *P* value from one-way ANOVA test is shown. The asterisks represent significant pairwise differences based on the Tukey test (*P* = 3.25e^−8^ between iBET151 and DMSO, *P* = 1.93e^−8^ between prostratin and DMSO, and *P* = 1.93e^−8^ between vorinostat and DMSO). **B.** Dot plots of RNA transcript levels for the regions of the HIV genome: LTR, Tat, Vpu, Env, and d2EGFP. **C.** UMAP plot of the scRNA-seq data color-coded based on the levels of HIV RNA expressions. **D.** Transformed total HIV RNA reads across different cell clusters. **E.** Coverage plots showing normalized read counts from scRNA-seq (left) and scATAC-seq (right) along the HIV genome for different cell clusters. The dark blue and gray boxes represent gene annotations and ATAC peaks. **F.** Dot plot showing ATAC-seq signals summed across the entire HIV genome for different clusters. **G.** HIV RNA expression in 2D10 cells is marginally correlated with HIV chromatin accessibilities. The bar plot shows the squared proportion of HIV RNA reads (Y-axis), stratified by the integer-valued total number of HIV ATAC read counts, which has a maximum of three due to the sparsity of scATAC-seq (X-axis). The *P* value was obtained from a Pearson correlation testing. LTR, long terminal repeat; VOR, vorinostat.

**Figure S5 HIV viral RNA expression and chromatin accessibility for primary cell donor 2**

**A.** Transformed HIV RNA reads under different treatment conditions. *P* values from one-way ANOVA test and pairwise comparison are shown. **B.** UMAP plot of the scRNA-seq data color-coded based on the levels of HIV RNA expression. **C.** Transformed HIV scATAC-seq reads under different treatment conditions. *P* values from one-way ANOVA test and pairwise comparison are shown. **D.** UMAP plot of the scATAC-seq data color-coded based on the levels of HIV mapping ATAC-seq reads. **E.** Correlation scatter plot showing the square root proportion of ATAC-seq reads mapping to HIV (X-axis) versus the square root proportion of RNA-seq reads mapping to HIV (Y-axis) across the cell population. **F.** Coverage plots showing normalized read counts from scATAC-seq along the HIV reference genome for different treatment conditions. The dark blue and gray boxes represent gene annotations and ATAC peaks respectively. **G.** Dot plots showing the percentage of cells expressing HIV and the average expression level for vRNA (left panel), and percent of cells with accessible HIV DNA and the average accessibility for different regions of the proviral genome (right three panels).

**Figure S6 2D10 cell cluster 6 is driven by HIV RNA expression**

**A.** Transformed total HIV RNA reads across different cell clusters. **B.** UMAP plots of scRNA-seq (left) and scATAC-seq (right) data. The colors represent treatment conditions (top), clusters (middle), and proportions of the HIV RNA expression from the full data analysis (bottom). **C.** Same as B except that the HIV genes were excluded for the clustering and UMAP construction.

**Figure S7 Cluster 7 & 8 are driven by HIV RNA expression for primary cell donor 1**

**A.** Transformed total HIV RNA reads across different cell clusters. **B.** UMAP plots of scRNA-seq (left) and scATAC-seq (right) data. The colors represent treatment conditions (top), clusters (middle), and proportions of the HIV RNA expression from the full data analysis (bottom). **C.** Same as B except that the HIV genes were excluded for the clustering and UMAP construction.

**Figure S8 Cluster 3 & 15 are driven by HIV RNA expression for primary cell donor 2**

**A.** Transformed total HIV RNA reads across different cell clusters. **B.** UMAP plots of scRNA-seq (left) and scATAC-seq (right) data. The colors represent treatment conditions (top), clusters (middle), and proportions of the HIV RNA expression from the full data analysis (bottom). **C.** Same as B except that the HIV genes were excluded for the clustering and UMAP construction.

**Figure S9 Differentially expressed genes induced by LRAs in 2D10 cells**

**A.** Venn diagrams showing the number of genes that were downregulated (the top panel) and upregulated (the bottom panel) upon treatment with LRAs (FDR adjusted *P* value 0.05). **B.** Heatmaps showing normalized and scaled RNA expression levels for different treatment conditions. The rows contain representative genes that were downregulated (left) and upregulated (right), respectively. The columns represent single cells.

**Figure S10 Differentially expressed genes induced by LRAs in donor 2 cells**

**A.** Venn diagrams showing the number of genes that were down-regulated (the top panel) and upregulated (the bottom panel) upon treatment with LRAs (FDR adjusted *P* value 0.05). **B.** Heatmaps showing normalized and scaled RNA expression levels for different treatment conditions. The rows contain representative genes that were downregulated (left) and upregulated (right), respectively. The columns represent single cells. **C.** *P* values from the differential expression analyses conducted in donor 1 and donor 2 exhibit a moderate correlation. The difference is likely due to donor-specific gene regulatory effects.

**Figure S11 Differentially accessible TFs following LRA stimulation of donor 2 cells**

**A**. Venn diagrams showing the number of TFs whose motif accessibilities were significantly decreased (the upper panel) or increased (the lower panel) upon treatment with LRAs compared to DMSO (FDR adjusted *P* value 0.05). **B.** Box plots showing distributions of motif accessibilities (deviation scores) of selected affected TFs with decreased accessibility scores (SNAI2, SREBF1, ZNF135) or increased accessibility scores (NFATC2, RBPJ, TEAD2). *P* values from Wilcoxon rank sum tests comparing DMSO and one of the LRA-treated samples were integrated using the Cauchy combination. **C.** Box plots showing distribution of motif accessibilities of positive controls, FOS and JUN.

**Figure S12 Differentially accessible TFs following LRA stimulation of 2D10 cells**

**A**. Venn diagrams showing the number of TFs whose motif accessibilities were significantly decreased (the left panel) or increased (the right panel) upon treatment with LRAs compared to DMSO (FDR adjusted *P* value 0.05). **B.** Violin plots showing distributions of motif accessibilities (deviation scores) of FOS and JUN. *P* values from Wilcoxon rank sum tests comparing DMSO and one of the LRA-treated samples were integrated using the Cauchy combination. **C.** Same as B but for the four intersected TFs from A: FIGLA, SNAI1, SNAI3, and SNAI2.

**Figure S13 Footprinting analysis on differentially accessible TFs following LRA stimulation**

**A.** Footprinting plots of four intersected TFs**:** FIGLA, SNAI1, SNAI3, and SNAI2 in the upper panel FOS and JUN in the lower panel from 2D10 cell line data. **B.** same as A for TFs: SNAI2, SREBF1(Var.2), ZNF135, JUN, NFATC2, RBPJ, TEAD2, and FOS from primary donor 1 data. **C.** Same as B but from primary donor 2 data.

**Figure S14 Linkage analysis between HIV viral expression and genes/TFs/peaks for 2D10 cell data**

**A.** Distributions of nominal *P* values from gene expression linkage analysis. **B.** Visualizations of top linked genes. The dotted line represents the fitted line from a simple regression; Spearman correlation coefficient and nominal *P* value are included in the title. **C.** Same as A but for TF activity linkage analysis. **D.** Same as B but for TF activity linkage analysis. **E.** Same as A but for peak linkage analysis. **F.** Visualization of motifs enriched in ATAC peaks that were positively (top) and negatively (bottom) associated with the HIV RNA expression.

**Figure S15 Linkage analysis between HIV viral expression and genes/TFs/peaks for primary cell donor 2**

**A.** Distributions of nominal *P* values from gene expression linkage analysis. **B.** Visualizations of selected linked genes. The dotted line represents the fitted line from a simple regression; Spearman correlation coefficient and nominal *P* value are included in the title. **C.** Venn diagrams showing the number of significant genes correlated with HIV gene overlapped for donor 1 and donor 2. **D.** Same as A but for TF activity linkage analysis. **E.** Same as B but for TF activity linkage analysis. **F.** Same as C for TF activity linkage analysis. **G.** Same as A but for peak linkage analysis. **H.** Visualization of motifs enriched in ATAC peaks that were positively (top) and negatively (bottom) associated with the HIV RNA expression. **I.** Same as C for motif enrichment analysis.

**Figure S16 Comparison of gene/TF-linkage analysis between the aggregated and condition-specific samples for 2D10 cell data**

**A.** Pairwise scatterplots comparing the square root of negative log-transformed nominal *P* values from tests for a non-zero slope in simple linear regression of the HIV expression on cellular gene expression between different treatment conditions. The combined *P* values were obtained from the samples treated with iBET151, prostratin, and vorinostat. **B.** Same as A but for TF motif scores. Prostrat, prostratin.

**Figure S17 Comparison of gene/TF-linkage analysis between the aggregated and condition-specific samples for primary cell donor 1**

**A.** Pairwise scatterplots comparing the square root of negative log-transformed nominal *P* values from tests for a non-zero slope in simple linear regression of the HIV expression on cellular gene expression between different treatment conditions. The combined *P* values were obtained from the samples treated with iBET151, prostratin, and vorinostat. **B.** Same as A but for TF motif scores.

**Figure S18 Comparison of gene/TF-linkage analysis between the aggregated and condition-specific samples for primary cell donor 2**

**A.** Pairwise scatterplots comparing the square root of negative log-transformed nominal *P* values from tests for a non-zero slope in simple linear regression of the HIV expression on cellular gene expression between different treatment conditions. The combined *P* values were obtained from the samples treated with iBET151, prostratin, and vorinostat. **B.** Same as A but for TF motif scores.

**Figure S19 TF regulators associated with HIV latency in 2D10 cells**

**A.** A Venn diagram showing the number of significant TFs from three different testing schemes: TF linkage analysis, enrichment analysis of TF-binding motifs using significantly linked peaks, and analysis of differential motif accessibility. **B.** Heatmap showing correlations between the TF pairs. The correlation is computed using the TF-specific consensus principal components, calculated using both the TF expression and its motif accessibility. **C.** TF regulatory network.

**Figure S20 TF regulators of HIV latency reversal in primary cell donor 2**

**A.** A Venn diagram showing the number of significant TFs from three different testing schemes: TF linkage analysis, enrichment analysis of TF-binding motifs using significantly linked peaks, and analysis of differential motif accessibility. **B.** Heatmap showing correlation between the TF pairs. The correlation is computed using the TF-specific consensus principal components, calculated using both the TF expression and its motif accessibility. **C.** TF regulatory network.

**Figure S21 Machine learning analysis of multiomic data from 2D10 cells**

**A**. ROC curve for a machine learning model of HIV expression using the single cell multiomic dataset. The boosting-based model was trained on a subset of the overall data, then tested on the remainder. Representative curves from the training (orange) and test (blue) sets are shown. AUC is labeled. **B.** Ranking of model features based on overall contribution to model performance. ROC, receiver operating characteristic.

**Figure S22 GATA3 and FOXP1 regulate HIV latency**

**A.** One week post nucleofection of sgRNAs, knockout of the targets was analyzed by western blot. **B.** Flow cytometry at baseline and after 24 h of prostratin stimulation. Error bars represent standard deviations, and *P* values displayed were determined by one-way ANOVA Tukey’s multiple comparisons test.

**Table S1 Cluster specific transcripts for 2D10 cell line data, primary cell donor 1, and primary cell donor 2 data**

**Table S2 GO term enrichment analyses for gene that were significantly up-regulated and down-regulated upon treatment with LRAs for 2D10 cell line data**

**Table S3 GO term enrichment analyses for gene that were significantly up-regulated and down-regulated upon treatment with LRAs for donor 1 primary cell data**

**Table S4 GO term enrichment analyses for gene that were significantly up-regulated and down-regulated upon treatment with LRAs for donor 2 primary cell data**

**Table S5 Top 20 genes/TFs/peaks/enriched motifs linked with HIV viral expression for 2D10 cell line data**

**Table S6 Identification of cellular transcripts that correlate with HIV RNA levels for 2D10 cell line data**

**Table S7 Top 20 genes/TFs/peaks/enriched motifs linked with HIV viral expression for primary cell donor 2**

**Table S8 Identification of cellular transcripts and DATFs that correlate with HIV RNA levels for donor 1 primary cells**

**Table S9 Identification of cellular transcripts and DATFs that correlate with HIV RNA levels for donor 2 primary cells**

**Table S10** **GO term enrichment analysis for transcripts that were significantly correlated with HIV RNA levels for 2D10 cell line data, primary cell donor 1 data, and primary cell donor 2 data**

**Table S11** **Ranked list of cellular features based on representation within the set of GOSDT models of HIV expression in primary CD4 T cells**

**Table S12** **Ranked list of cellular features based on importance for XGBoost model of high HIV expression (top 10%) in primary CD4 T cells**
