# Supplementary figures and images for: Integrated Single-cell Multiomic Analysis of HIV Latency Reversal Reveals Novel Regulators of Viral Reactivation
Source: Genomics Proteomics Bioinformatics. 2024 Jun 20;22(1):qzae003. doi: 10.1093/gpbjnl/qzae003 (PMC11189801; doi:10.1093/gpbjnl/qzae003)

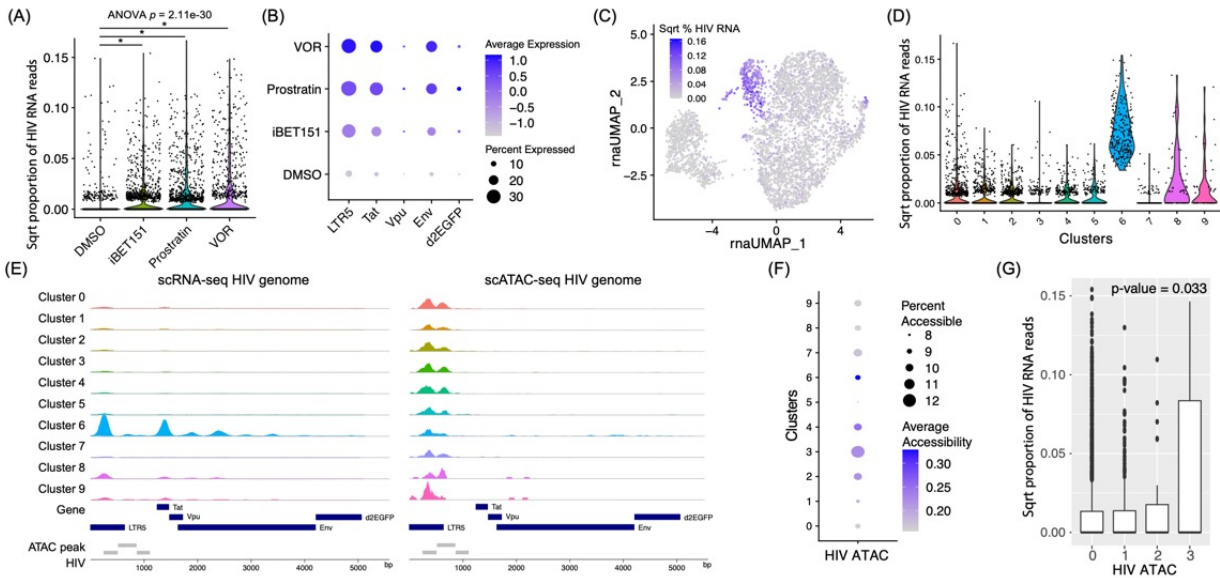

Supplement: qzae003_Supplementary_Data [file qzae003_supplementary_data.zip › Figure S4.pdf]

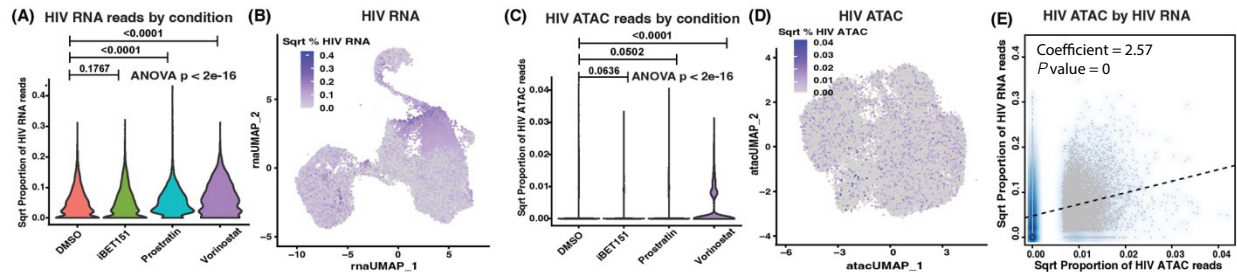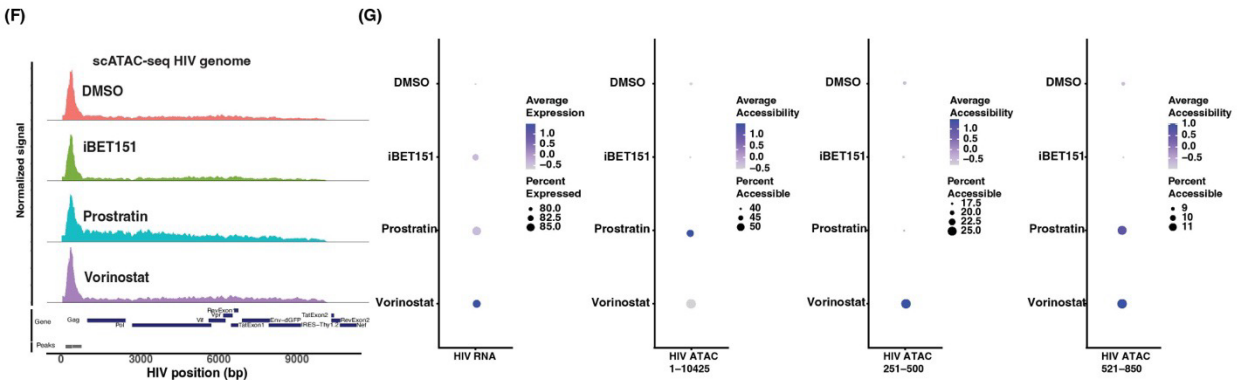

Supplement: qzae003_Supplementary_Data [file qzae003_supplementary_data.zip › Figure S5.pdf]

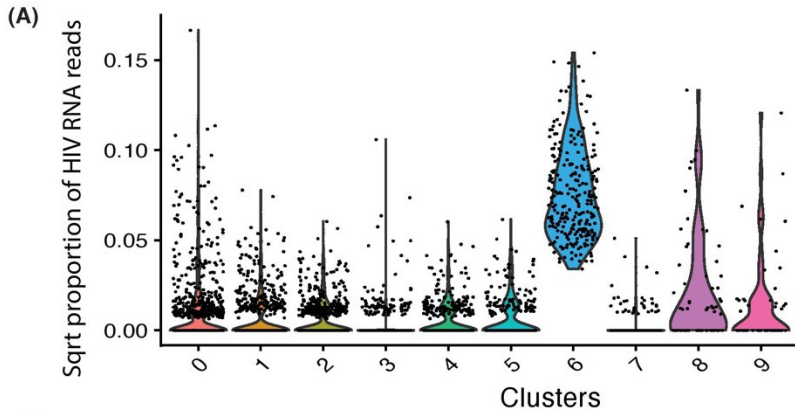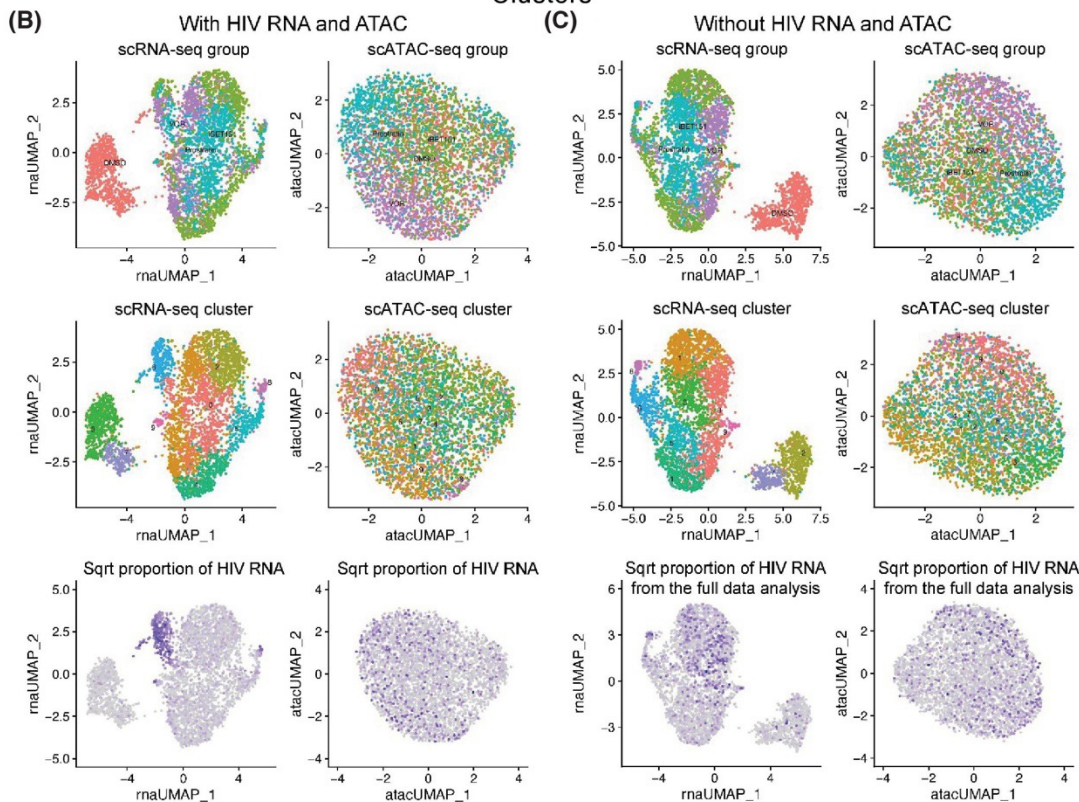

Supplement: qzae003_Supplementary_Data [file qzae003_supplementary_data.zip › Figure S6.pdf]

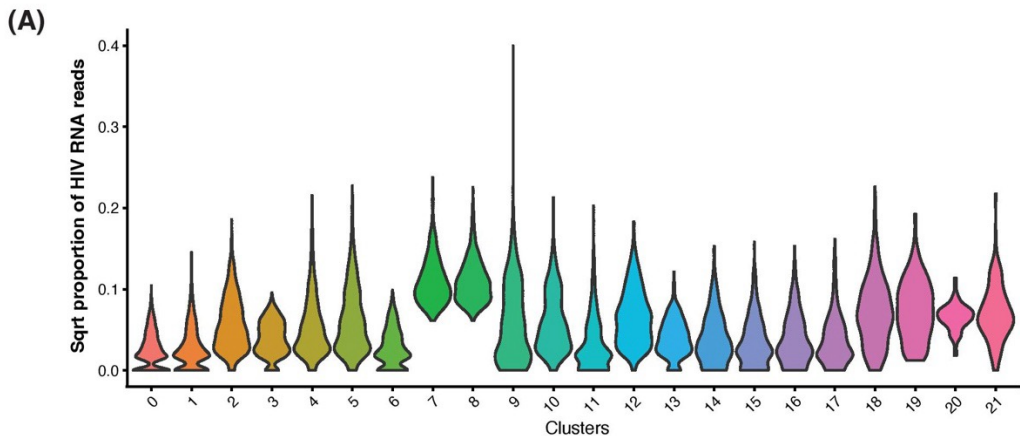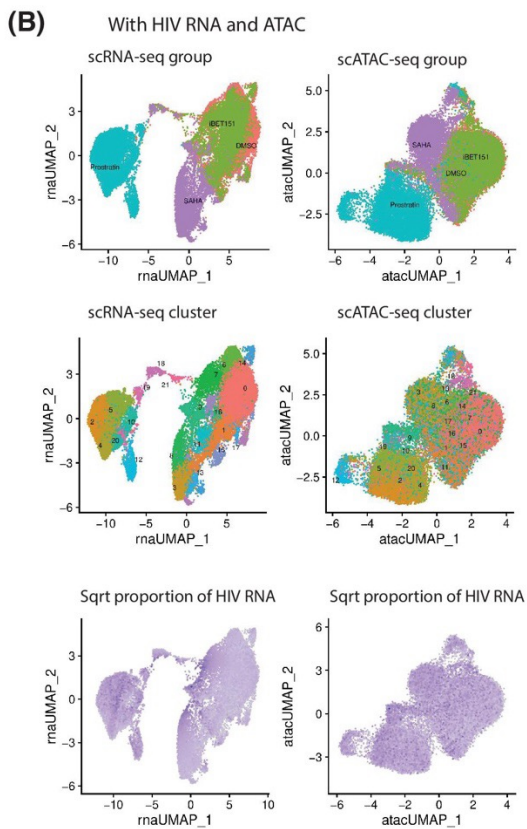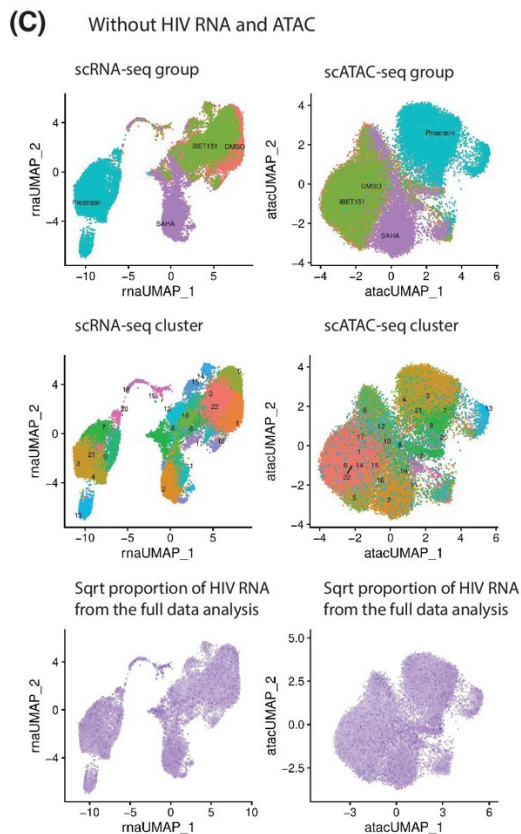

Supplement: qzae003_Supplementary_Data [file qzae003_supplementary_data.zip › Figure S7.pdf]

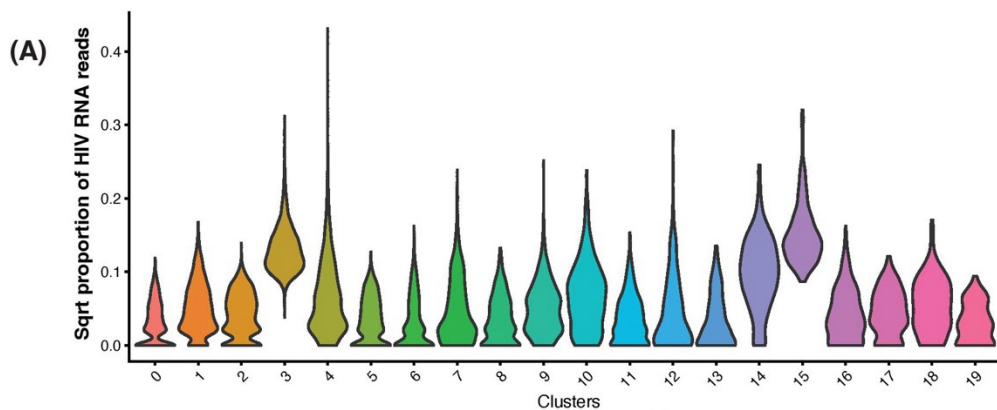

**(B)** With HIV RNA and ATAC

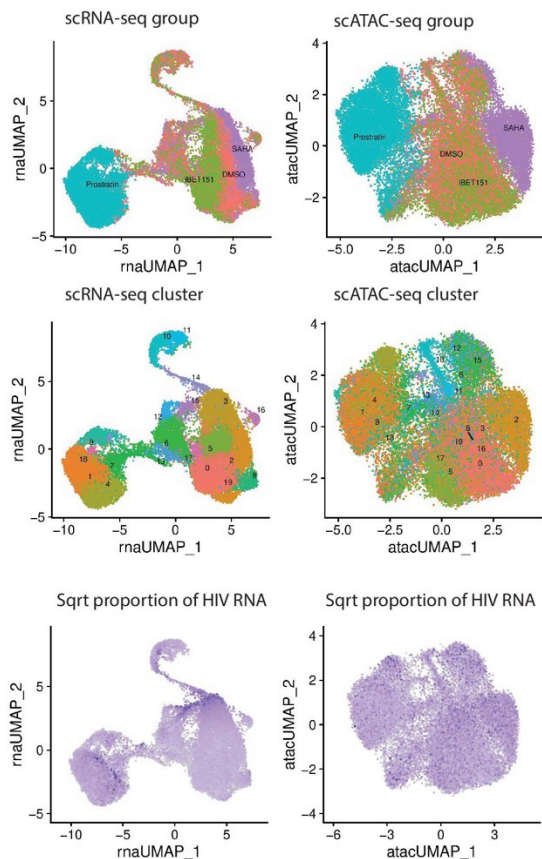

**(C)** Without HIV RNA and ATAC

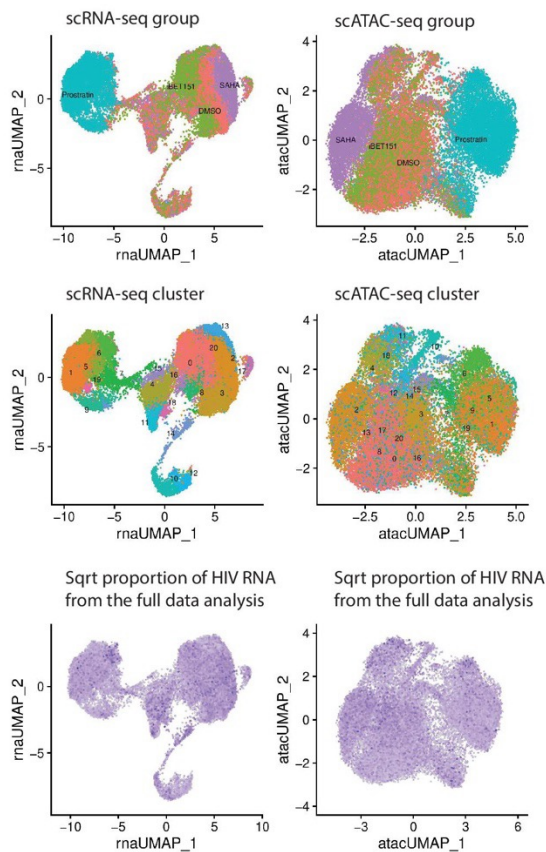

Supplement: qzae003_Supplementary_Data [file qzae003_supplementary_data.zip › Figure S8.pdf]

**(A)**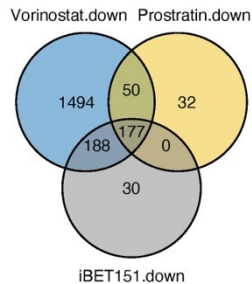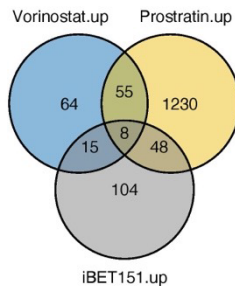**(B)**

Genes downregulated by LRAs

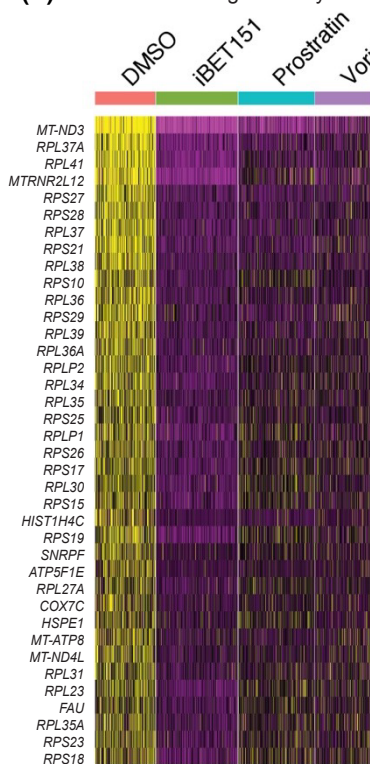

Genes upregulated by LRAs

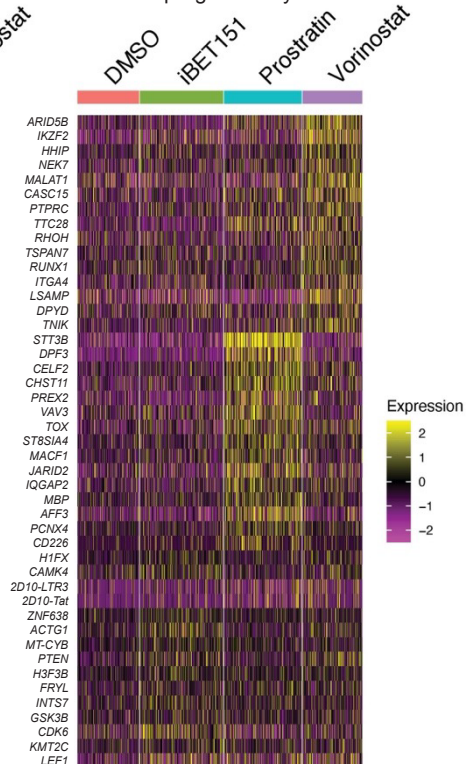

Supplement: qzae003_Supplementary_Data [file qzae003_supplementary_data.zip › Figure S9.pdf]

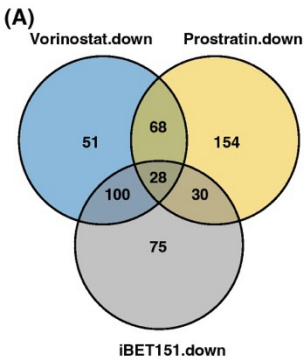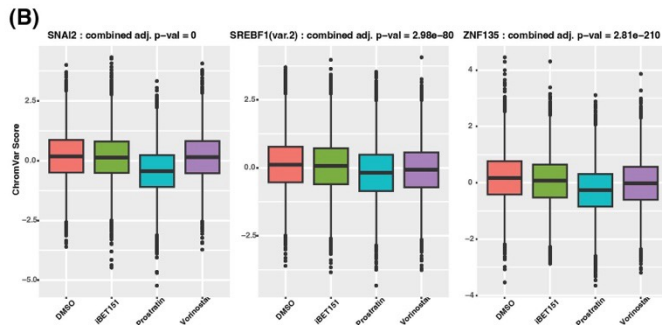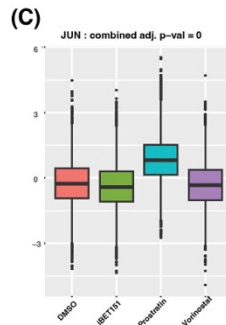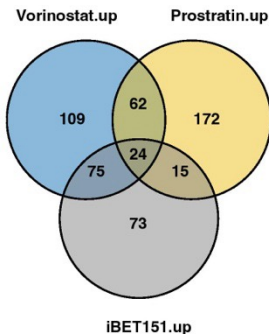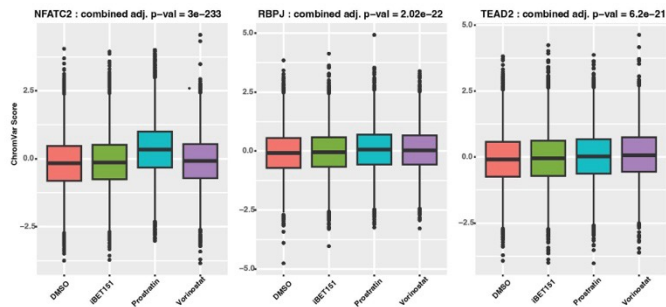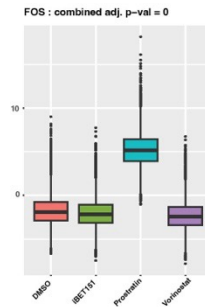

Supplement: qzae003_Supplementary_Data [file qzae003_supplementary_data.zip › Figure S11.pdf]

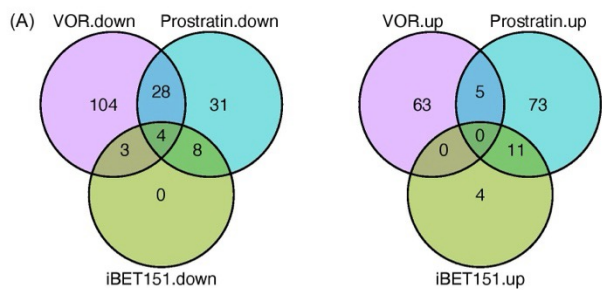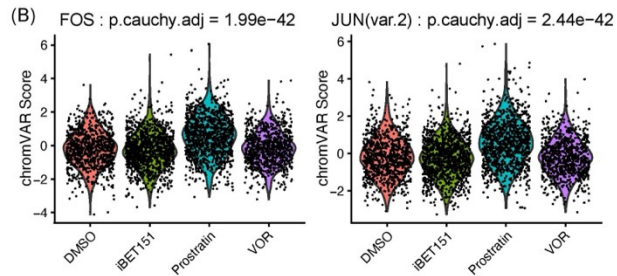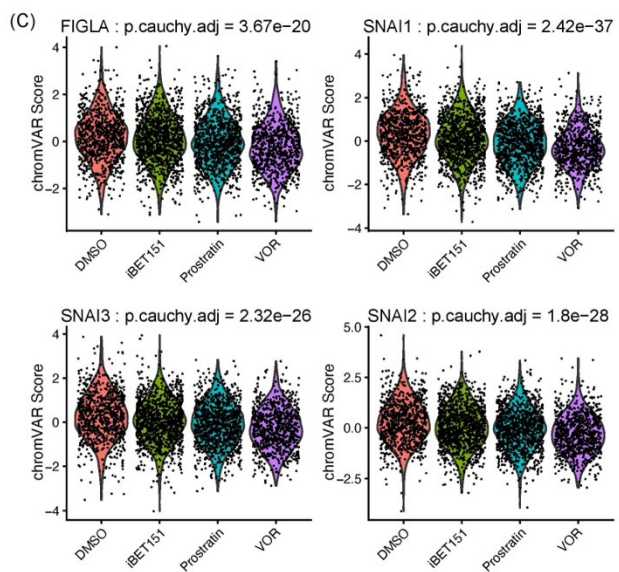

Supplement: qzae003_Supplementary_Data [file qzae003_supplementary_data.zip › Figure S12.pdf]

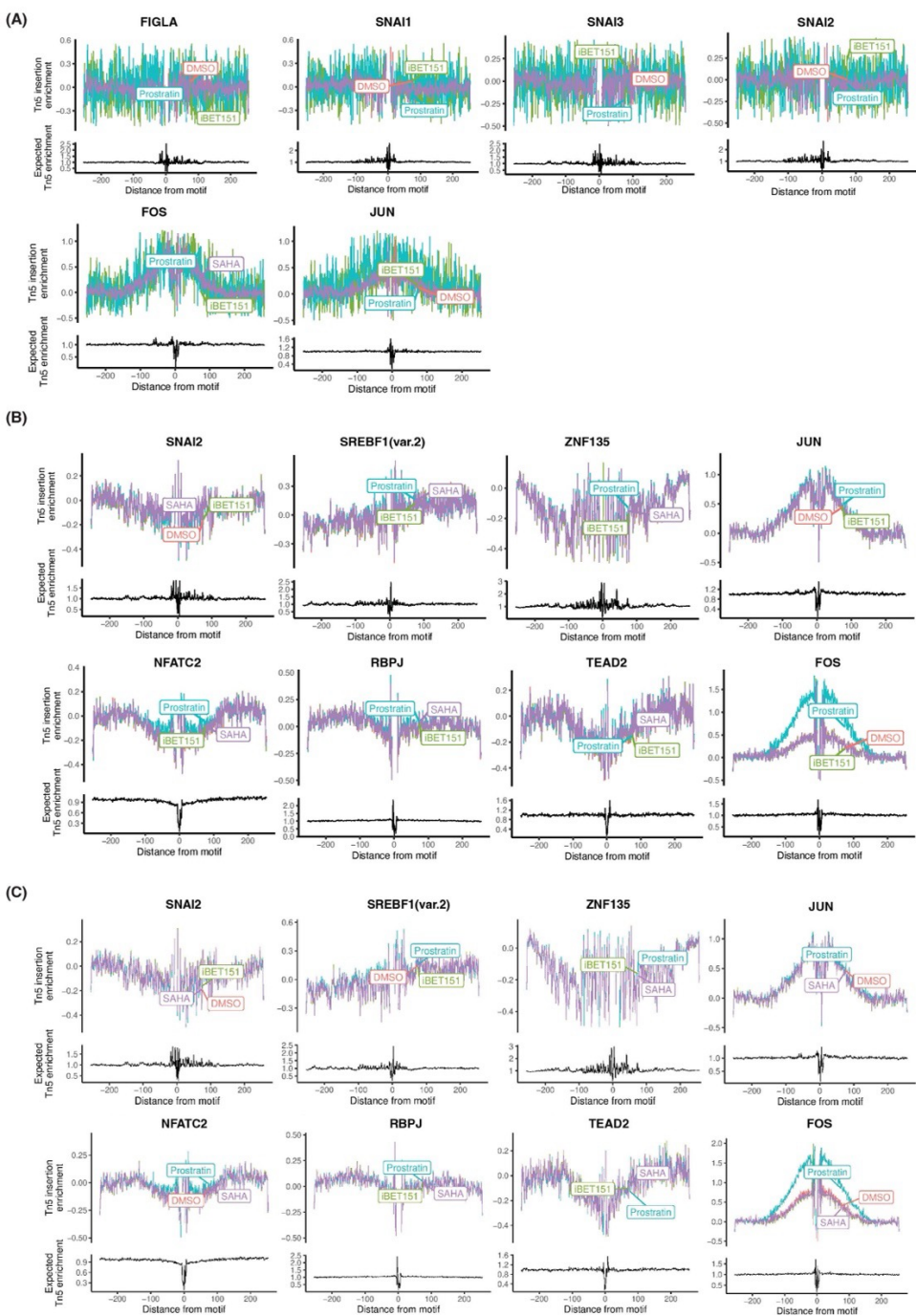

Supplement: qzae003_Supplementary_Data [file qzae003_supplementary_data.zip › Figure S13.pdf]

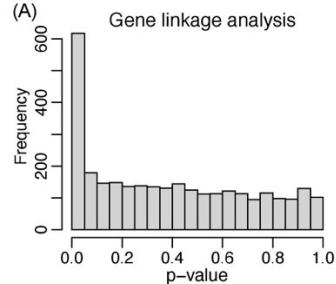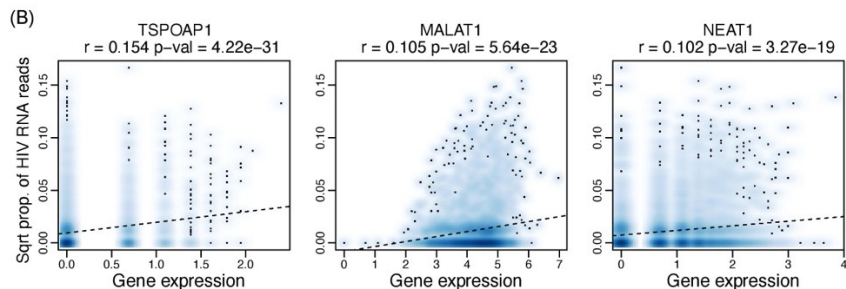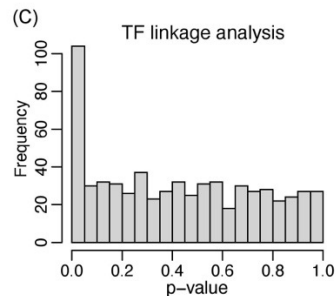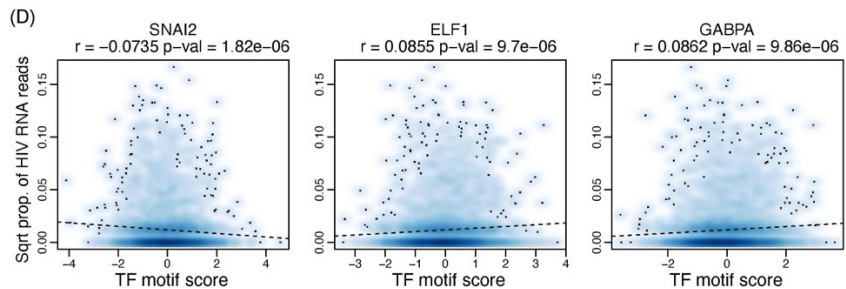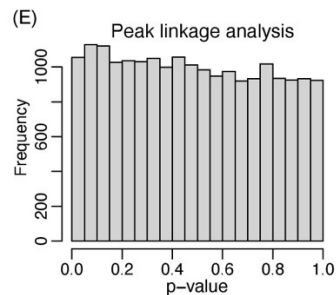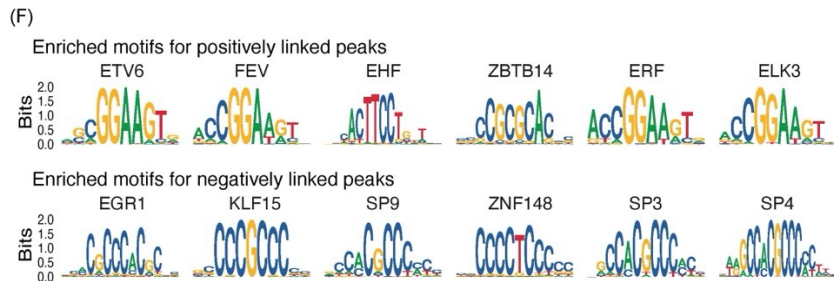

Supplement: qzae003_Supplementary_Data [file qzae003_supplementary_data.zip › Figure S14.pdf]

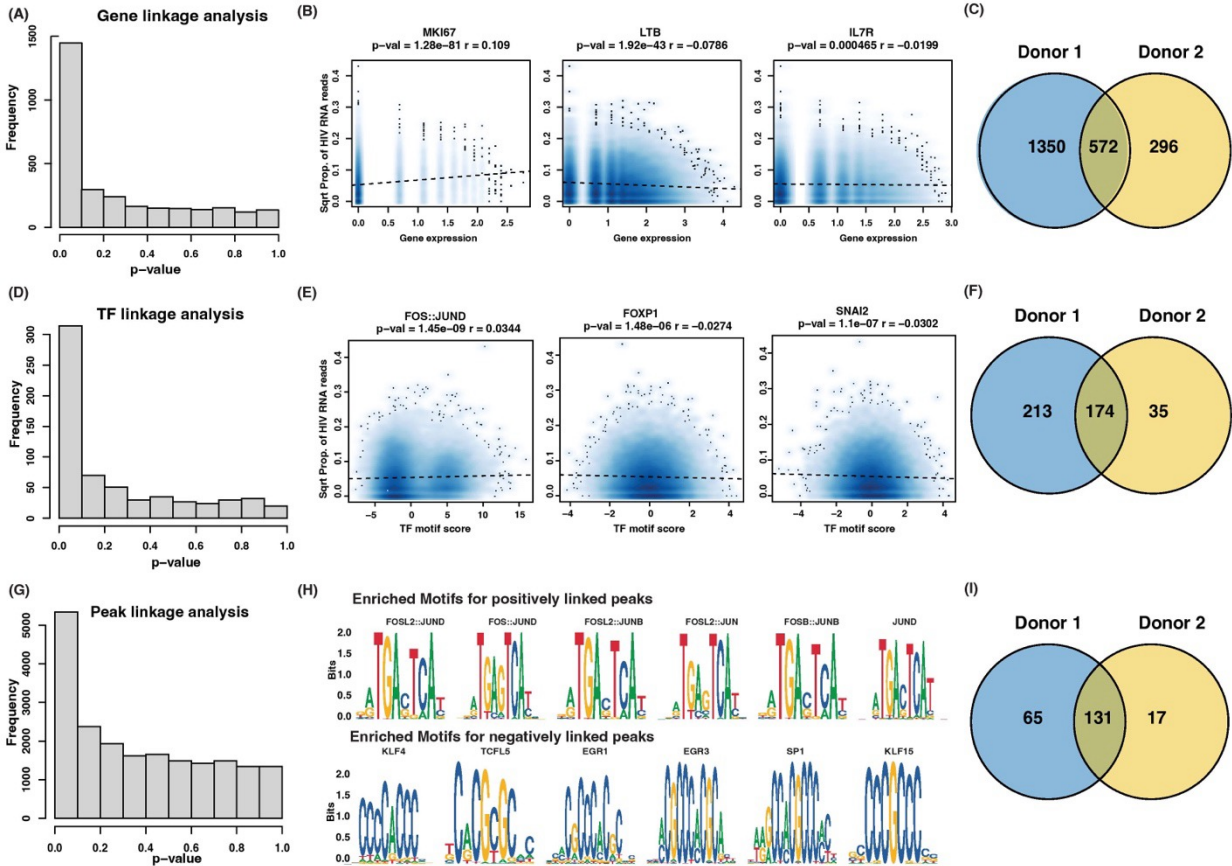

Supplement: qzae003_Supplementary_Data [file qzae003_supplementary_data.zip › Figure S15.pdf]

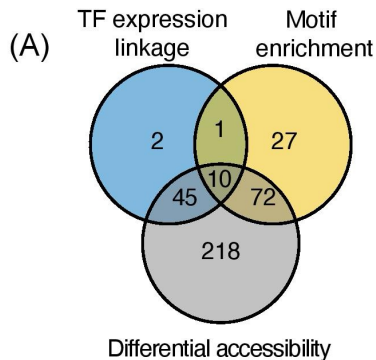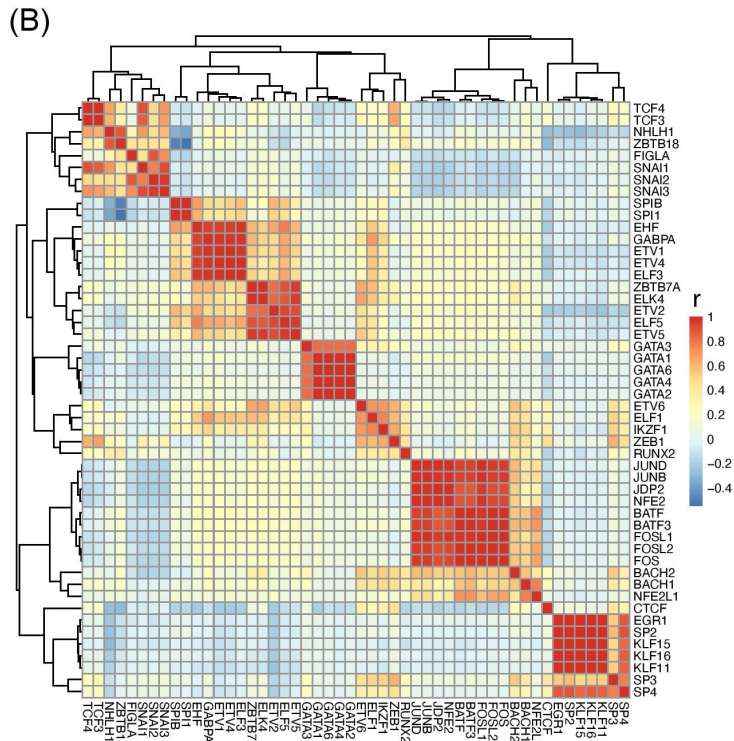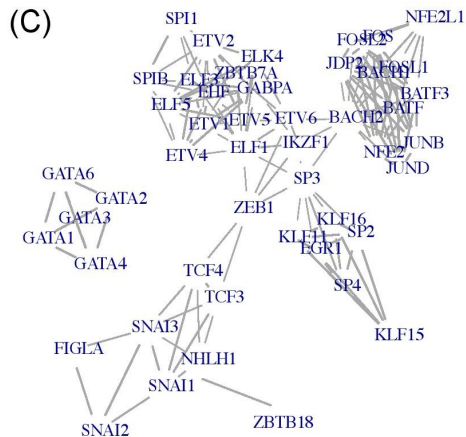

Supplement: qzae003_Supplementary_Data [file qzae003_supplementary_data.zip › Figure S19.pdf]

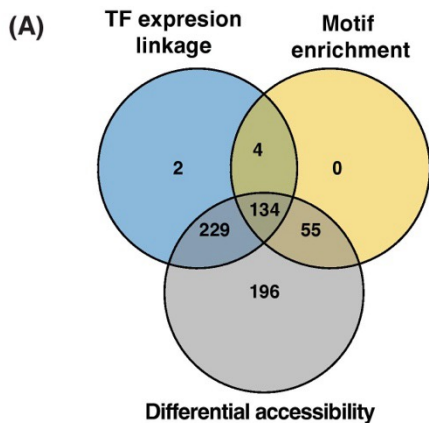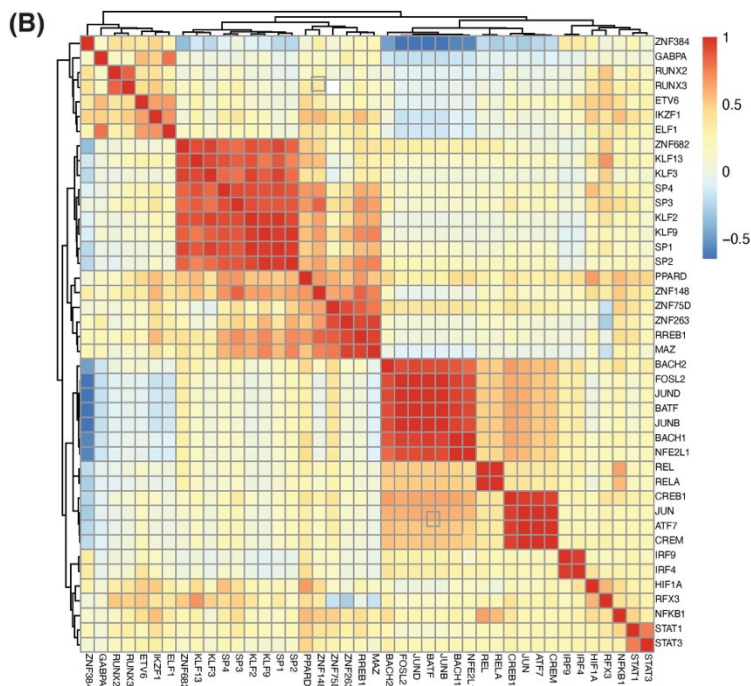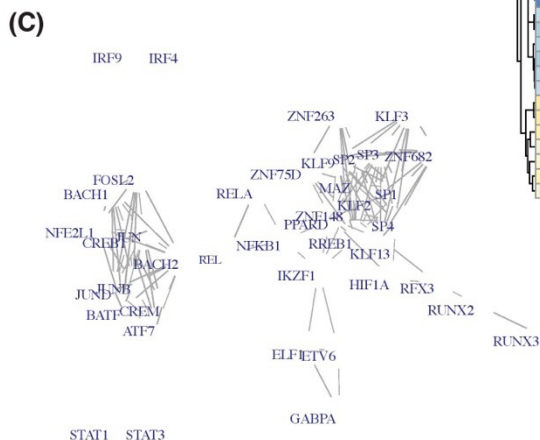

Supplement: qzae003_Supplementary_Data [file qzae003_supplementary_data.zip › Figure S20.pdf]

(A)

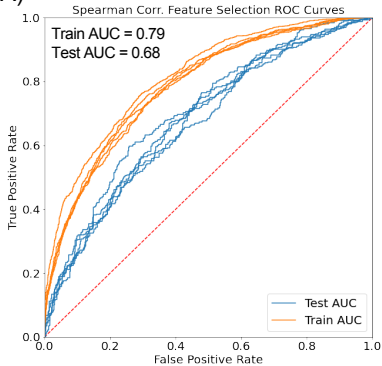

(B)

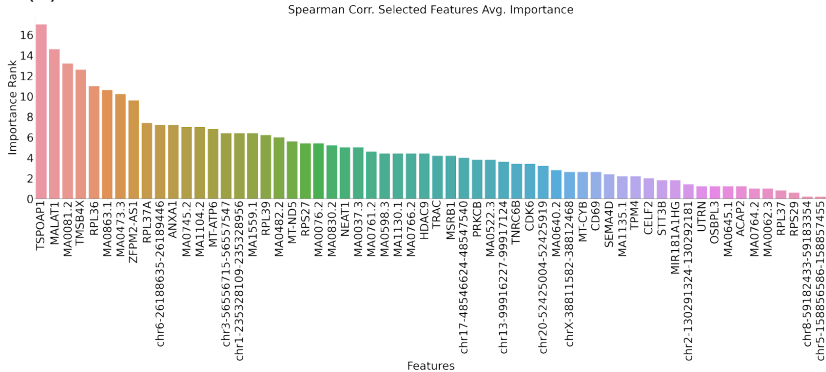

Supplement: qzae003_Supplementary_Data [file qzae003_supplementary_data.zip › Figure S21.pdf]

(A)

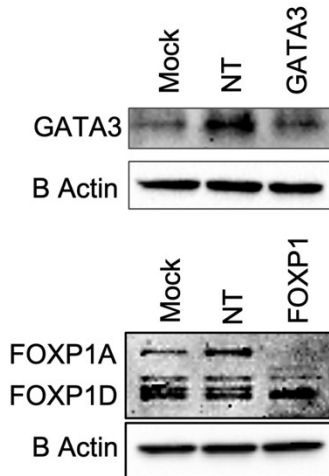

(B)

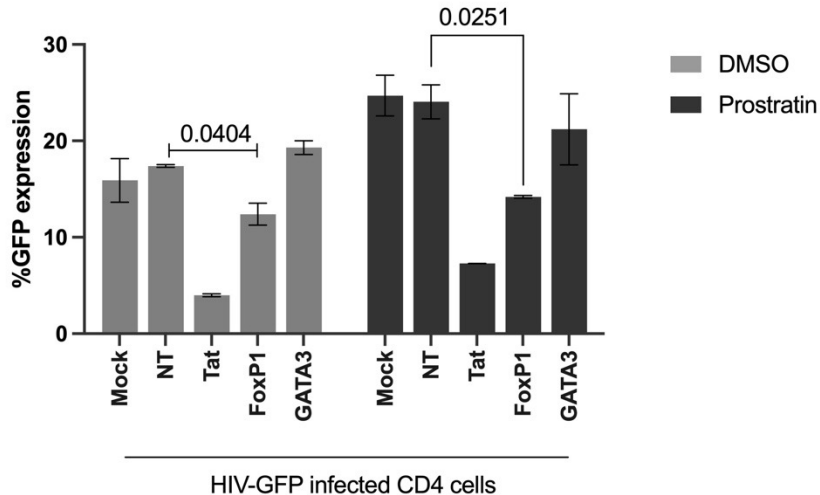

Supplement: qzae003_Supplementary_Data [file qzae003_supplementary_data.zip › Figure S22.pdf]

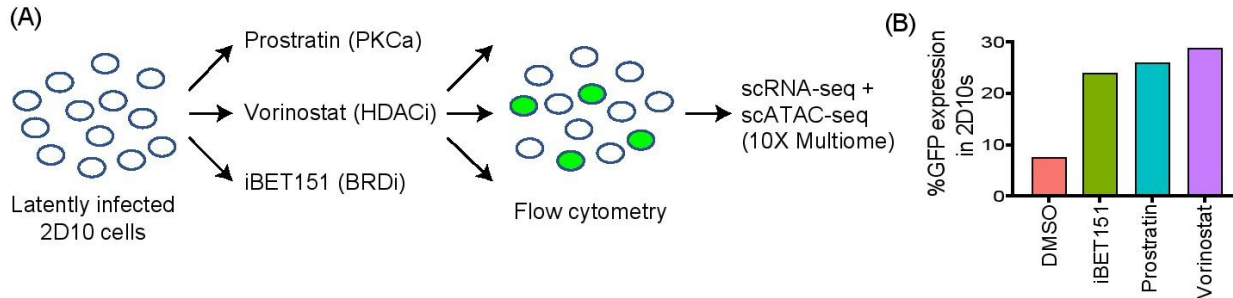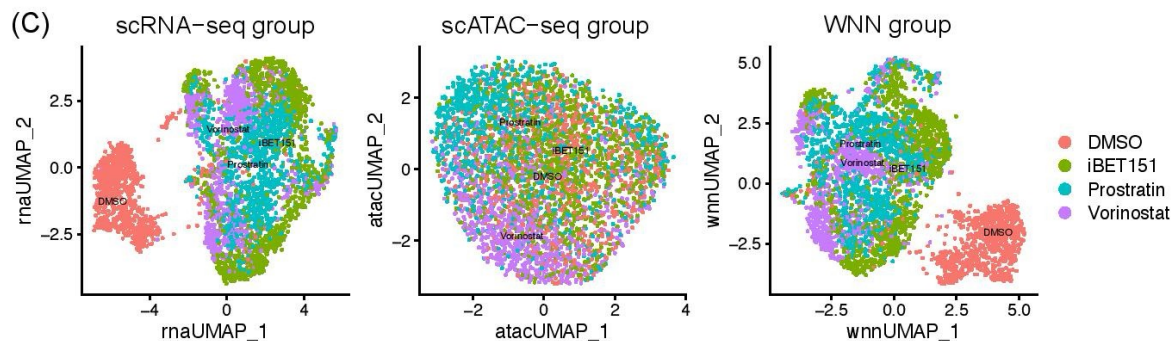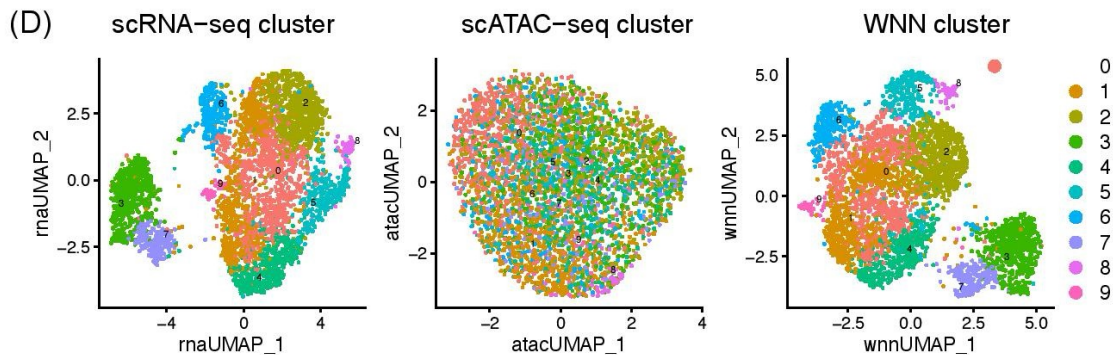

Supplement: qzae003_Supplementary_Data [file qzae003_supplementary_data.zip › Figure S1.pdf]

(A)

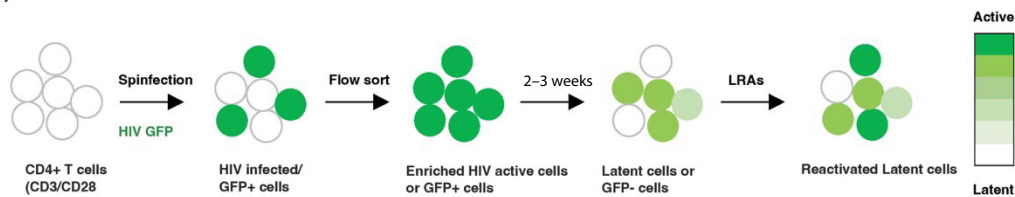

(B)

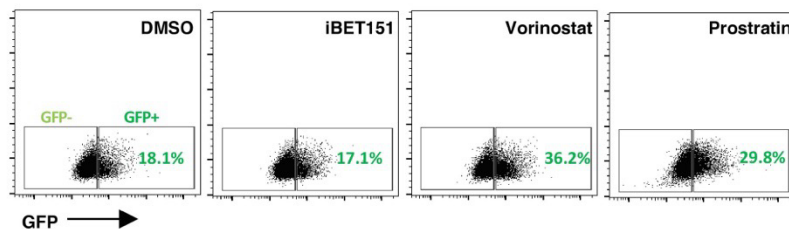

(C)

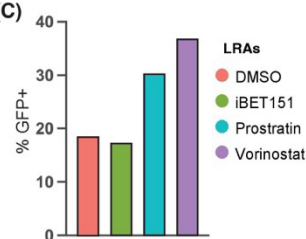

(D)

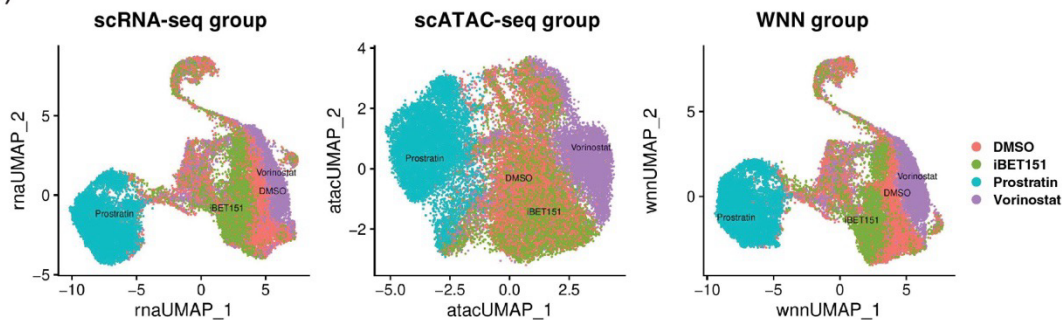

(E)

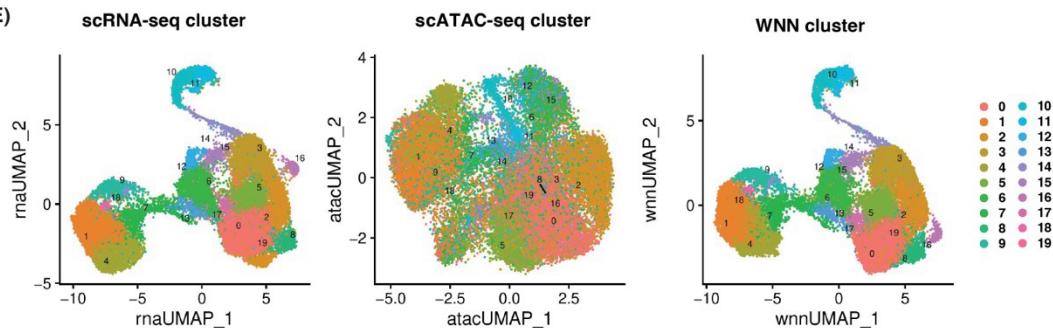

Supplement: qzae003_Supplementary_Data [file qzae003_supplementary_data.zip › Figure S2.pdf]

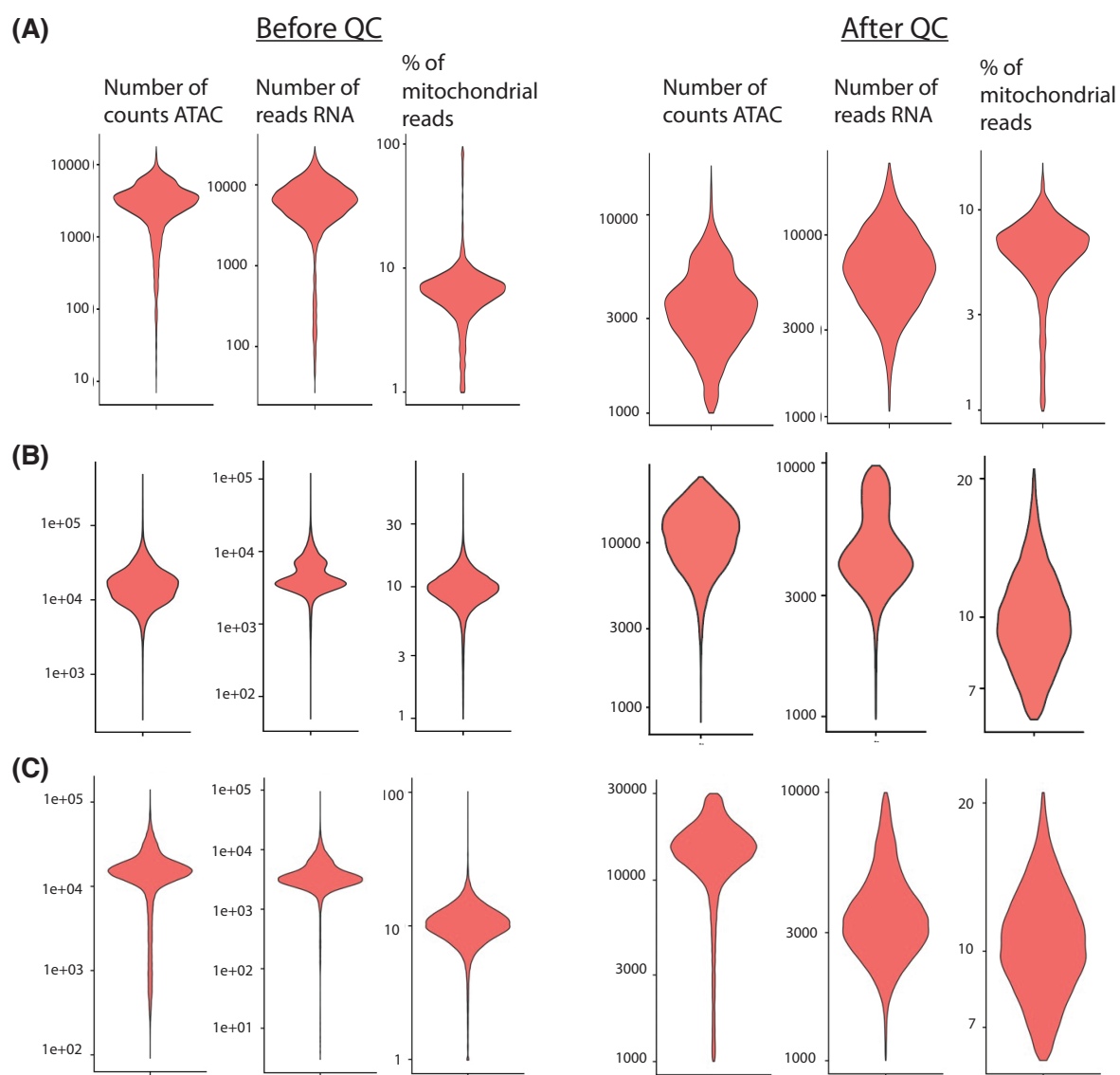

Supplement: qzae003_Supplementary_Data [file qzae003_supplementary_data.zip › Figure S3.pdf]
